# Wood Formation under Severe Drought Invokes Adjustment of the Hormonal and Transcriptional Landscape in Poplar

**DOI:** 10.3390/ijms22189899

**Published:** 2021-09-13

**Authors:** Dade Yu, Dennis Janz, Krzysztof Zienkiewicz, Cornelia Herrfurth, Ivo Feussner, Shaoliang Chen, Andrea Polle

**Affiliations:** 1 Beijing Advanced Innovation Center for Tree Breeding by Molecular Design, College of Biological Sciences and Technology, Beijing Forestry University, Beijing 100083, China; lschen@bjfu.edu.cn; 2 Forest Botany and Tree Physiology, Büsgen-Institute, University of Goettingen, 37077 Göttingen, Germany; djanz@gwdg.de; 3 Institute of Chinese Materia Medica, China Academy of Chinese Medical Sciences, Beijing 100700, China; 4 Department of Plant Biochemistry, Albrecht-Von-Haller Institute, University of Goettingen, 37077 Göttingen, Germany; krzysztof.zienkiewicz@biologie.uni-goettingen.de (K.Z.); cherrfu@uni-goettingen.de (C.H.); ifeussn@uni-goettingen.de (I.F.); 5 Service Unit for Metabolomics and Lipidomics, Göttingen Center of Molecular Biosciences (GZMB), University of Goettingen, 37077 Göttingen, Germany; 6 Department of Plant Biochemistry, Göttingen Center of Molecular Biosciences (GZMB), University of Goettingen, 37077 Göttingen, Germany

**Keywords:** drought, abscisic acid, secondary cell walls, phytohormone, transcriptional regulation

## Abstract

Drought is a severe environmental stress that exerts negative effects on plant growth. In trees, drought leads to reduced secondary growth and altered wood anatomy. The mechanisms underlying wood stress adaptation are not well understood. Here, we investigated the physiological, anatomical, hormonal, and transcriptional responses of poplar to strong drought. Drought-stressed xylem was characterized by higher vessel frequencies, smaller vessel lumina, and thicker secondary fiber cell walls. These changes were accompanied by strong increases in abscisic acid (ABA) and antagonistic changes in salicylic acid in wood. Transcriptional evidence supported ABA biosynthesis and signaling in wood. Since ABA signaling activates the fiber-thickening factor NST1, we expected upregulation of the secondary cell wall (SCW) cascade under stress. By contrast, transcription factors and biosynthesis genes for SCW formation were down-regulated, whereas a small set of cellulose synthase-like genes and a huge array of genes involved in cell wall modification were up-regulated in drought-stressed wood. Therefore, we suggest that ABA signaling monitors normal SCW biosynthesis and that drought causes a switch from normal to “stress wood” formation recruiting a dedicated set of genes for cell wall biosynthesis and remodeling. This proposition implies that drought-induced changes in cell wall properties underlie regulatory mechanisms distinct from those of normal wood.

## 1. Introduction

Wood is an important commodity for construction materials, biofuels, and as a feedstock for cellulose production [1,2]. Wood (botanically: xylem) is formed by the secondary growth of stems of trees. However, tree growth is severely constrained by harsh environmental conditions such as drought [3,4]. In order to reduce water loss and acclimate to drought, several physiological changes occur, including stomatal closure, reductions in photosynthetic CO_2_ assimilation, leaf area reduction, shoot growth cessation, leaf desiccation and abscission [5,6]. As a result, plant height and stem diameter growth are impeded and the aboveground biomass production is diminished. Unlike the aboveground responses, root growth is often maintained or even enhanced when sensing drought to adjust the uptake of dwindling water resources [7].

A further consequence of drought stress is the acclimation of the xylem architecture [8]. In angiosperms, the xylem is composed of vessels, fibers, and parenchyma cells. These cell types are formed during secondary growth of the stem, starting from the cambial zone with cell division, expansion, differentiation, lignification and ending with programmed cell death (PCD) in the mature xylem [9,10]. Water and mineral nutrients absorbed by roots are transported via vessels through the xylem, while structural support of the plant is provided by the fibers [11]. Vessels and fibers are characterized by distinct lumina and secondary cell walls (SCWs) [12], composed of lignin, cellulose, hemicellulose and small amounts of pectin and proteins [13]. Under drought, new xylem cells with thicker walls are formed and the vessels are narrower and more abundant compared to unstressed wood [11,14,15,16].

In recent years, much progress has been made in our understanding of the molecular regulation of wood formation [17]. The regulation of the processes leading to the specification of xylem cells and apposition of secondary cell walls is very complex and highly dynamic and, thus, not entirely understood. A working model suggests that a transcriptional cascade consisting of three layers of transcription factors (TFs) governs SCW formation from the initiation to the biosynthesis of lignin, cellulose, and hemicelluloses [18,19]. This model is continuously refined because of new discoveries of interacting factors and control loops [20,21], but there is agreement that several *VASCULAR RELATED NAC DOMAIN* (*VND1*–*VND7*) TFs are vessel-specific and spatially and temporally expressed in tight correlation with xylem cell differentiation [22]. Another group of NAC TFs consisting of *NAC SECONDARY WALL THICKENING PROMOTING FACTOR1* (*NST1*), *NST2* and *SECONDARY WALL-ASSOCIATED NAC-DOMAIN 1* (*SND1/NST3*) is responsible for the initiation of SCW formation, particularly in the process of *Arabidopsis* fiber cell wall thickening [23,24,25]. The VNDs and NSTs are placed tentatively at the top of the transcriptional cascade as master regulators (1st level). The expression of these master regulators is modulated by the HD-Zip transcription factors or VND-INTERACTION 2 (VNI2) [26,27], which are fine-tuning factors. TFs from the MYB family are regulated by the master regulators of the 1st level and constitute two further hierarchical levels (2nd and 3rd level regulators). In *Arabidopsis*, MYB46 and MYB83 are functioning as the second level regulators, initiating SCW development by orchestrating other MYBs and TFs on the third level. Among TFs on the third level, the expression of *MYB20*, *MYB42*, *MYB43*, *MYB52*, *MYB54*, *MYB69*, *MYB85*, *MYB103*, *SND2*, and *SND3* affect the structure and composition of secondary cell walls, regulating the expression of genes involved in biosynthesis of cellulose, hemicelluloses, and lignin [28,29]. In contrast to the TFs promoting the expression of genes involved in the biosynthesis of SCW, constituents of the third level, *MYB75* and *KNAT7* repress gene expression for hemicellulose synthesis [30,31]. The TFs *MYB4*, *MYB7,* and *MYB32* inhibit the expression of *NST3/SND1* on the first level [32,33] and form a negative-feedback loop. The transcriptional regulation of SCW biosynthesis known for *Arabidopsis* was shown to be partially conserved in tree species such as *Populus* sp. [34]. However, knowledge on the impact of drought on the regulatory network of the *Populus* orthologs expressed during wood formation is scarce. Therefore, an important goal of this study was to investigate the response of the SCW regulatory network to drought stress under well-characterized physiological conditions.

Phytohormones also play very important roles in wood formation, regulating cambium activity, initiating xylem cell differentiation, and mediating stress responses [35,36,37,38,39,40]. Among various phytohormones coordinating plant development (auxin, cytokinins, brassinosteroids, gibberellines, ethylene), abscisic acid (ABA) is prominent for its role in regulating stress acclimation [41]. ABA regulates leaf stomatal closure, leads to delayed leaf development, and enhanced primary root elongation [42,43,44,45]. In poplar xylem, ABA is an abundant hormone (*P. nigra*, *P.* × *canescens* [46,47]). Transgenic modifications in ABA signaling exert drastic effects on leaf size and poplar biomass production [48]. In *Arabidopsis*, ABA defective mutants exhibit delayed fiber production [49]. Furthermore, the ABA signaling pathway is involved in activation of VNDs and NST1, the 1st level master TFs required for xylem differentiation or SCW formation in *Arabidopsis* [50,51]. These findings imply that a coordinated regulation of ABA signaling together with the SCW TF cascade is required for stress acclimation. However, data are lacking to underpin this hypothesis.

Plant stress adaptation is regulated by hormone-induced signaling cascades [52]. Synergistic interactions have been postulated for ABA and JA [53] but whether they mediate adaptive stress responses in wood is less understood. In contrast to relatively limited knowledge on ABA and JAs in wood formation, the role of auxin has been intensely studied for a longtime (e.g., [54,55,56,57]). Auxin provides positional information [55] and is a key factor for growth promotion via control of cambial activity by AUXIN RESPONSIVE FACTORs (ARFs) [58]. In salt-stressed wood, auxin levels decrease together with reduction vessel diameters [36]. It is unknown how stress-induced changes in wood anatomy are related to shifts in auxin and JA/ABA levels.

To study the principles of wood formation, the genus of *Populus* is used as a model organism because of its rapid growth, ease of clonal propagation, amenability for genetic transformation, and its economic relevance [59]. Hybrids of *P. tremula* (European species) and the closely related north American species *P. tremuloides* show great promise for forest plantations [60]. To promote the domestication of these hybrids, we need to understand their stress tolerance. Here, we used the well-established clone T89 (*P. tremula* × *tremuloides*) for the investigation of drought responses in the xylem. The main aim of this study was to uncover co-regulation of phytohormones with changes in xylem anatomy and master regulators of SCW under drought.

## 2. Results

### 2.1. Phytohormone Profiling Detects Tissue-Specific Drought Responses Concurring with Physiological Stress Adaptation

**Figure 1:**
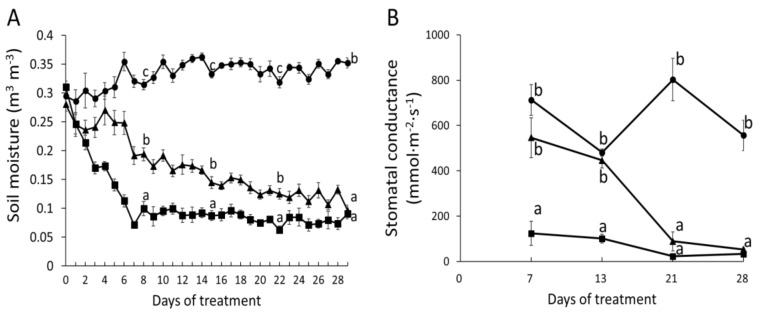
Soil moisture in pots (**A**) and stomatal conductance of poplar (hybrid T89) (**B**) in response to moderate (triangles) and severe (squares) drought stress and well-watered (circles) conditions. (**A**) Soil moisture in pots of plants in response to drought. Data show means ± SE (*n* = 8). One-way ANOVA was conducted with the data measured on 8th, 15th, 22nd, and 29th day of the treatment. Tukey-test was applied post-hoc and means that differ at *p* ≤ 0.05 are indicated by different letters. (**B**) Stomatal conductance of plants in response drought. Data show means ± SE (*n* = 8) measured on 7th, 13th, 21th, and 28th day of the treatment. One-way ANOVA was conducted for each day. Tukey-test was applied post-hoc and means that differ at *p* ≤ 0.05 are indicated by different letters.

**Figure 2:**
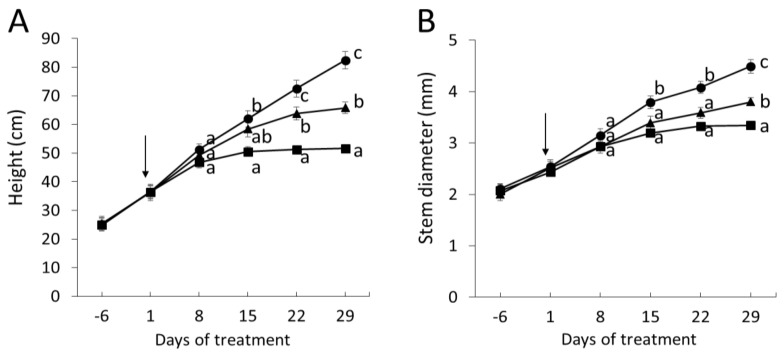
Height (**A**) and diameter (**B**) growth of poplar (hybrid T89) in response to moderate (triangles) and severe (squares) drought stress and well-watered (circles) conditions. Data show means ± SE (*n* = 8). One-way ANOVA was conducted with the data measured on the 8th, 15th, 21st, and 28th day of the treatment. Tukey-test was applied post-hoc and means that differ at *p* ≤ 0.05 are indicated by different letters. Arrows mark the start of the drought treatment.

To investigate the physiological responses to severe drought, we withheld water until the soil moisture declined from control levels of 0.3–0.4 m^3^ m^−3^ of the well-watered poplars to values below 0.1 m^3^ m^−3^ (Figure 1A). Subsequently, the plants were kept at low soil moisture (0.085 ± 0.002 m^3^ m^−3^) by controlling the amounts of added water. Severe drought caused a drastic decline in stomatal conductance already after one week (Figure 1B). For comparison, a moderate drought regime, which resulted in significant effects on stomatal conductance after three weeks is also shown (Figure 1A,B). Under severe stress, stem height and diameter growth were significantly reduced after two weeks of drought in comparison with well-watered controls (Figure 2A,B). Under moderate stress, stem height and diameter growth assumed an intermediate position between controls and severely stressed plants (Figure 2A,B).

The growth reductions of drought-stressed poplars were accompanied by reductions in the formation rate of new leaves, decreased leaf size and thus, massive reductions in whole-plant leaf area and biomass (Table 1). Since root biomass was not significantly reduced under drought, severely stressed poplars showed an increased root-to-shoot ratio (Table 1).

We conducted phytohormone profiling in leaves, wood, and fine roots of well-watered and drought-stressed poplars. Among eight compounds analyzed, six showed significantly different concentrations among the tissues but only three [ABA, ABA-glucose ester (ABA-GE), salicylic acid (SA)] showed significant drought effects (Table 2). The contents of the growth hormone indolic acetic acid (IAA) and the stress hormone JA were quite variable and therefore, the IAA decline (−27%) and JA increase (+2.8-fold) in drought-stressed wood were not significant at *p* < 0.05 (Table 2). Still, these changes may be biologically relevant. Among the drought-responsive phytohormones, SA increased in roots (Table 2).

The most profound drought effects were found for ABA. ABA accumulated to high concentrations in wood of severely stressed plants (Table 2). ABA levels were generally much higher in wood and leaves than in roots (Table 2). ABA-GE was highest in leaves (Table 2). These observations suggest that excess ABA synthesized in response to drought was stored in its inactive form ABA-GE in leaves. In contrast to leaves, the ABA-GE concentrations in wood of stressed poplars were about ten-fold lower than the free ABA levels and increased moderately in response to drought (Table 2).

### 2.2. Wood Anatomical Characteristics Are Strongly Changed in Response to Severe Drought

**Figure 3:**
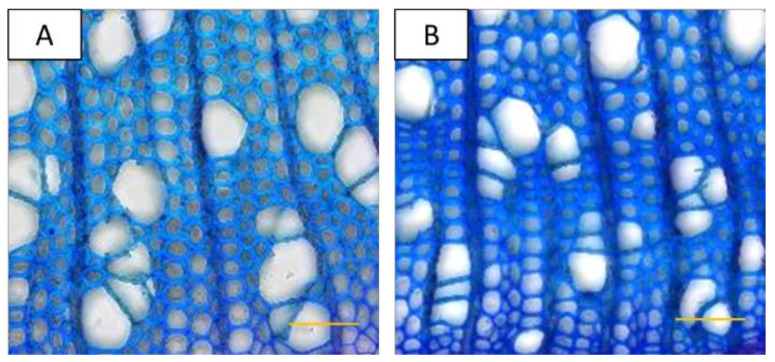
Cross sections of poplar (hybrid T89) wood from well-watered (**A**) and severely drought-stressed (**B**) plants. 400× magnification, bar = 50 µm, staining: toluidine blue.

We inspected wood formed during drought stress in comparison with that of well-watered plants in cross sections taken close to the stem base (Figure 3). The vessel frequency of drought-stressed plants was approximately two-fold higher and the vessel lumina approximately two-fold smaller than those of well-watered plants (Table 3). The fiber frequency varied considerably in drought-stressed wood and therefore, no significant effect (at *p* < 0.05) was observed compared to controls. The fiber lumina were smaller (−34%) and the fiber cell walls were thicker (+11%) in drought-stressed than in well-watered plants (Table 3). The reduction in fiber lumina was caused by increases in cell wall thickness (Table 3). A further consequence of decreased vessel and fiber lumina and increased cell wall thickness was an increase in the relative fraction of cell wall area per cross sectional area in xylem of stressed compared to unstressed plants (Table 3). However, the total cell wall area of xylem produced during the stress treatment was strongly reduced (−58%) in comparison with that produced by well-watered plants (Table 3). This reduction was the result of the growth decrement caused by an approximately two-fold reduction in the cambial cell layers of the stressed plants (Table 3), indicating an impaired cambial activity under severe drought.

### 2.3. Drought Stress Reprograms the Wood Transcriptome

RNA sequencing was conducted to investigate the changes of transcript abundances in the xylem of plants exposed to severe drought stress compared with controls. Among the 27,707 genes composing the whole transcriptome detected in wood, 13,234 differentially expressed genes (DEGs at *p*_adj_ < 0.05, Bonferroni correction) were identified, of which 6808 were up-regulated and 6426 were down-regulated (Supplement Table S1,see Suplementary Materials). Thus, half of the detected genes were affected by water shortage, indicating a strong transcriptional regulation of plant adaptation to severe drought stress.

**Figure 4:**
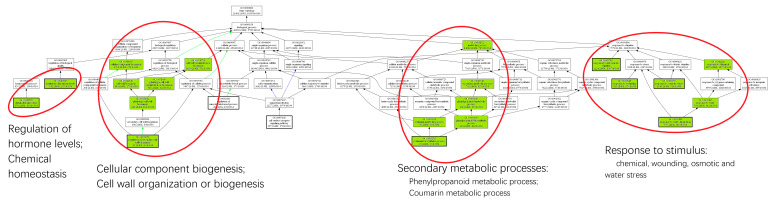
Hierarchy of enriched GO-terms in wood of poplar (hybrid T89) in response to severe drought. DEGs were used to determine significant GO terms. Enriched GO terms (*p*_adj_ < 0.05) are colored in green and GO terms not enriched are shown in white. Some GO terms were omitted in this figure. Further details are shown in Supplement Figure S1.

The DEGs were classified according to Gene Ontology (GO) terms, revealing 63 significantly (*p*_adj_ < 0.05) enriched GO terms (Supplement Table S2). Among them, 37 were assigned to the category “biological process”, 16 to “molecular function” and 10 to “cellular component”. GO terms in the category “biological process” revealed that drought stress influenced the transcription of genes involved in regulating hormone levels, water stress responses, secondary metabolic processes, as well as the biogenesis of cellular constituents such as cell walls (Figure 4). A closer inspection, differentiating between positively and negatively regulated DEGs showed that upregulated DEGs were enriched in “response to ABA”, “response to wounding” (including jasmonate-related genes), and “positive regulation of flavonoid biosynthesis” (Supplement Figure S1A). The down-regulated DEGs were enriched in GO terms for “growth”, “cytoskeleton and microtubuli organization”, “xylem development”, “cell wall biogenesis and organization”, “lignin metabolism”, and “cellulose biosynthesis” (Supplement Figure S1B). This indicated that suppression of xylem development and secondary cell wall formation was linked with activation of ABA responses.

### 2.4. Transcriptional Regulation of ABA and Other Phytohormones in Xylem under Severe Drought

**Figure 5:**
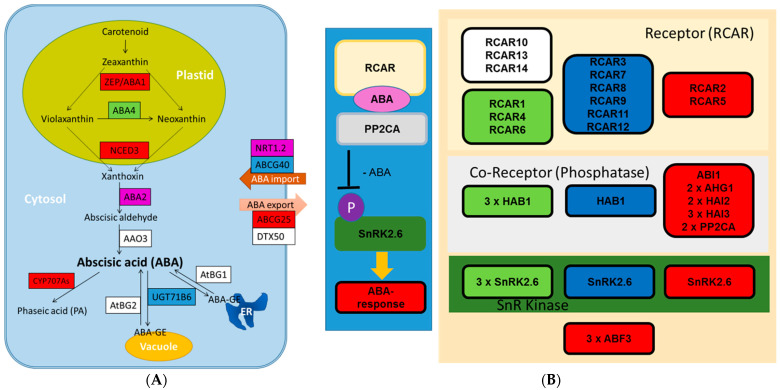
Transcriptional regulation of ABA metabolism (**A**) and ABA core signaling (**B**) in wood of poplar (hybrid T89) in response to severe drought stress. (**A**) Overview on ABA metabolism (modified after Chen et al. [68]) indicating drought-regulated genes in poplar xylem. The data for the transcriptional responses are shown in Supplemental Table S3. Color code: White: gene not detected. Green: gene not significantly affected. Red: gene significantly up-regulated. Darkblue: gene significantly down-regulated. Magenta: poplar homologs show contrasting responses. Please note that poplar often contains several homologs that match one *Arabidopsis thaliana* locus. Abbreviations: ZEP, zeaxanthin epoxidase; NCED, 9-cis-epoxycarotenoid dioxygenase; AAO, abscisic aldehyde oxidase; BG, β-glucosidases. (**B**) ABA core signaling involving the ABA receptors (RCARs, yellow box), ABA-coreceptors (phosphatase, grey box), SnRK2.6 kinases (green box) and down-stream ABA-responsive transcription factors (red box). Color code for genes in the boxes is the same as in (**A**). The 14 RCAR genes in poplars were named as suggested by Papacek et al. [61]. Other genes were poplar homologs to the corresponding *Arabidopsis* genes.

Under drought, the genes composing the pathways for ABA biosynthesis and ABA signaling showed strong transcriptional regulation (Figure 5A,B, Supplement Table S3). Genes for enzymes of ABA biosynthesis localized in plastids, *ABA1* and *NCED3* exhibited significantly increased transcript abundances (Figure 5A, Supplement Table S3). Especially, the two homologs of *NCED3* were more than 16-fold overexpressed (Supplement Table S3), suggesting continued activation of ABA biosynthesis in stressed xylem, even though the drought treatment had lasted for already four weeks. A puzzling observation was that transcripts of the ortholog to *AtAAO3* were not detected. Blasting the *Arabidopsis AAO3* nucleotide sequence in the *P. trichocarpa* v.3 Phytozome (https://phytozome.jgi.doe.gov (accessed on 3 April 2021)) picked Potri.004G191300.1 and Potri.009G153800.1 as closest homologs. Transcripts for these poplar genes were either significantly down-regulated or unaffected in our study. Inactivation of ABA might have chiefly been reached by ABA degradation or export since *CYP707A* (ABA hydroxylation) and *ABCG25* (transport) orthologs were upregulated, while the transcription of *UGT71B6* (glycosylation) and *ABCG40* (potential ABA import) were downregulated (Figure 5A, Supplement Table S3). Overall, the transcriptional changes in stressed xylem concur with the observed elevated ABA and low ABA-GE concentrations in wood (Table 2).

The main components of ABA signaling are RCARs (Regulatory Component of ABA Receptors, 14 members in poplar [61]), group A PP2Cs (type 2C protein phosphatases) and SnRK2 (sucrose non-fermenting 1-related protein kinase2) [62,63,64]. Among the main components implicated in ABA core signaling, most of the *PP2CA* homologs to *Arabidopsis* were up-regulated, whereas the majority of *RCAR* genes were down-regulated in stressed wood (Figure 5B). However, *RCAR2*, the most strongly expressed *RCAR* in non-stressed wood (corresponding to *Arabidopsis RCAR1/PYL9*), showed 2.5-fold increased transcript levels in response to drought (Figure 5B, Supplement Table S3). The *SnRK2* transcript levels were not or only slightly affected in response to drought stress (Figure 5B). However, SnRK2.6s are post-translationally regulated by phosphorylation: in the absence of stress when ABA levels are low, PP2Cs dephosphorylate SnRK2s and suppress their activities [62]. When ABA levels increase under stress, RCARs bind ABA and PP2As, forming a self-inactivating complex [63,64], thereby, enabling SnRK2 phosphorylation (Figure 5B). Then, SnRK2 actives downstream transcription factors (TFs), such as ABFs (ABA-responsive element binding factors) [65]. Here we found up-regulation of three *ABF3* orthologs in stressed wood (Figure 5B, Supplement Table S3). Our data show that all genes required for ABA biosynthesis are present in poplar wood and the ABA signaling cascade is activated in drought-stressed xylem.

In addition to ABA, we investigated transcriptional changes for the genes involved in JA and IAA biosynthesis in drought-stressed wood. The biosynthesis of IAA starts in chloroplasts with formation of tryptophan from chorismate, which is converted in the cytosol to indole-3-acetic acid via indole-3-pyruvate [66]. The regulation of different steps showed a heterogeneous pattern but the final step, which is catalyzed by YUCCA (a flavin-containing monooxygenase) was transcriptionally significantly down-regulated in drought-stressed compared to well-watered plants (Supplement Figure S2A, Table S4).

The biosynthetic pathway of JA [67] also showed significantly changed transcript abundances in the wood of drought-stressed compared to the well-watered trees. While some key steps were upregulated, especially the expression of *FATTY ACID DESATURASE 8* (*FAD8*) at the entrance of the pathway, other steps were represented by several homologs with contrasting responses (Supplement Figure S2B, Table S4). The induction of allene oxide synthase is a marker for JA biosynthesis but its poplar homologs showed contrasting transcriptional regulation (Supplement Figure 2B). It is possible that severe drought resulted in precarious conditions, causing an unstable state for JA production.

### 2.5. The Transcriptional Cascade Governing Secondary Cell Wall Formation Is Suppressed by Drought

**Figure 6:**
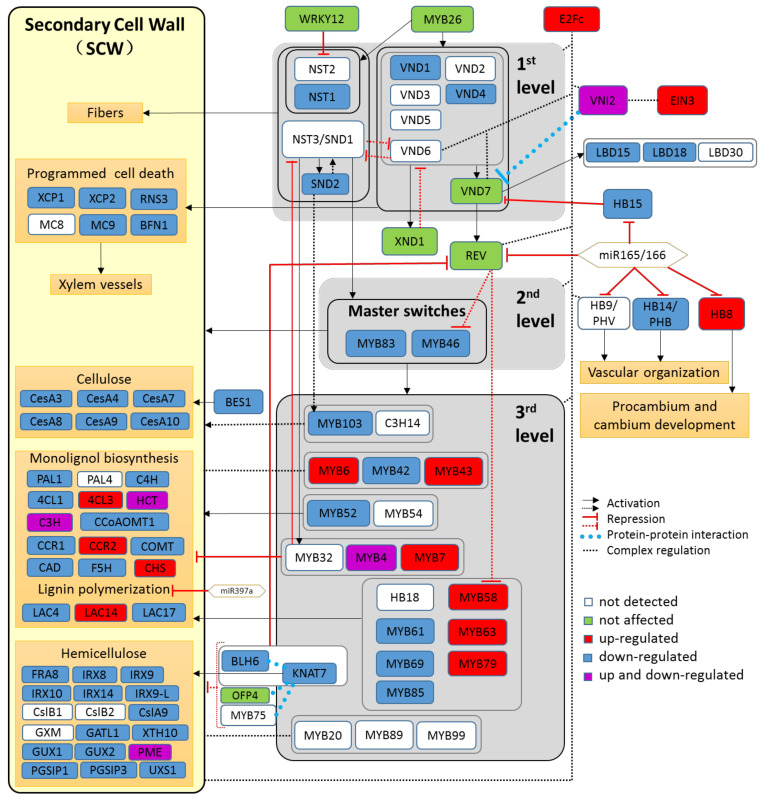
Transcriptional regulation of genes for secondary cell wall formation in poplar (hybrid T89) wood in response to severe drought stress. The cell wall signaling cascade was modified after Zhang et al. [19] and Hussey et al. [71] and shows the poplar homologs to *Arabidopsis* genes. Color code: White: genes not detected. Green: genes not significantly affected. Red: genes significantly up-regulated. Darkblue: genes significantly down-regulated. Magenta: poplar homologs show contrasting responses. Please note that poplar often contains several homologs that match one *Arabidopsis thaliana* locus. The data are shown Supplemental Table S4.

Since the fibers in stressed poplar xylem have increased cell wall thickness (Table 3), we expected that drought would induce the SCW transcriptional cascade and activate the transcription of genes required for the production of cell wall constituents such as cellulose, hemicellulose, and lignin. However, none of the 1st and 2nd level TFs of the SCW cascade were upregulated (Figure 6, data in Supplement Table S5). The fine-tuning factors, homologs of *AtHB15* as well as one homolog of *VNI2* were down-regulated, whereas the other two homologs of *AtVNI2* were strongly up-regulated (Figure 6). VNI2 inhibits VND7 and negatively regulates vessel formation [26], but the poplar homologs of *AtVND7* were not differentially expressed under drought stress (Figure 6).

**Figure 7:**
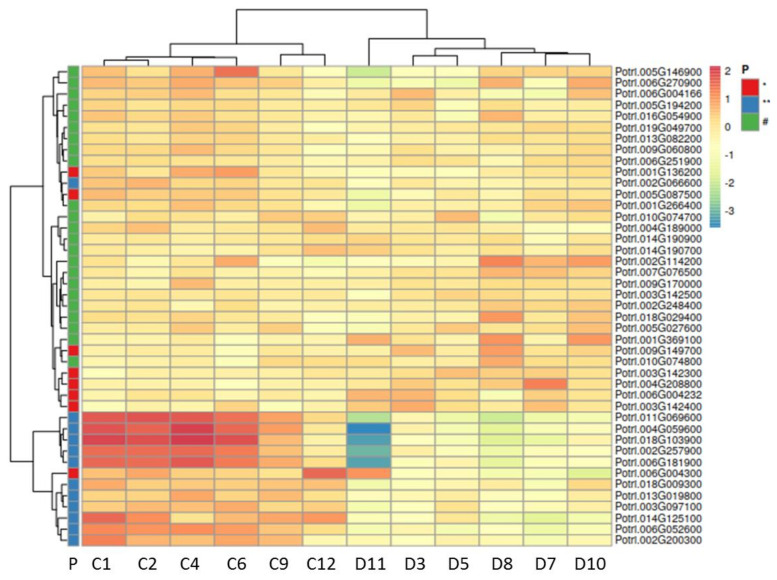
Hierarchical cluster analysis of genes related to cellulose synthesis in poplar wood (hybrid T89) of drought-stressed and non-stressed plants. Transcript abundances of genes annotated as “cellulose synthase” and “cellulose synthases-like” were retrieved from Supplement Table S1, and subjected to cluster analysis after transformation [ln(x + 1)] using Ward and Euclidian distance. P indicates *p*-values for the comparison of means of drought-stressed with non-stressed plants: * *p* < 0.05, ** *p* < 0.01, # not significant. C1, C2, C4, C6, and C9 are control samples and D11, D3, D5, D8, D7, and D10 are samples collected from drought-treated plants. Two main clusters were formed. The cluster on the top (31 annotated genes) was not or weakly upregulated in drought-stressed poplars. Transcript abundances of these genes were low. The lower cluster (12 annotations) was strongly expressed in non-stressed wood and massively downregulated in wood in response to drought stress. The heatmap was drawn using ClustVis [72].

The 3rd level TFs directly regulate genes governing lignin, cellulose, and hemicellulose biosynthesis [28,29]. Distinct members of gene families encoding enzymes for the production lignin precursors and laccase exhibited increased transcript abundances in drought-stressed xylem (Figure 6, data in Supplement Table S6), while most poplars orthologs of the *Arabidopsis* genes involved in cellulose and hemicellulose biosynthesis showed strong reductions in transcript abundances (Figure 6, data in Supplement Table S7). This result was unexpected since cell wall thickness is due to apposition of cellulose and hemicelluloses, while incorporation of lignin makes the wall more hydrophobic and rigid. Since cell wall biosynthetic genes are members of strongly expanded families in poplar compared with *Arabidopsis* [69,70], we extracted all genes with the annotation “cellulose synthases” and “cellulose synthase-like” from our data set (Supplement Table S1) and visualized their transcript abundances in a heat map (Figure 7). The heat map shows a clear separation of the expression pattern of the putative cellulose synthases in stressed compared with unstressed xylem (Figure 7). Twelve of 43 genes were highly expressed in poplar wood without stress and strongly downregulated under drought (Figure 7). A small cluster of four or five members exhibited low expression under control conditions and moderate increases under drought (Figure 7).

All detected poplar orthologs of *Arabidopsis* genes related to hemicellulose formation were down-regulated (Figure 6). However, a heatmap for all genes in our data set annotated as expansins, expansin-like genes, galacturonosyltransferases, galacturonosyltransferase-like genes, pectin methyl esterases, and xyloglucan endotransglucosylases/hydrolases in the transcriptome (Supplement Table S1) showed that their transcriptional responses were less succinct than of those for cellulose synthases, resulting in less clear separation of drought- and unstressed xylem (Supplement Figure S3). This result was also supported by MapMan categorization of the DEGs (Supplement Table S7). Among all DEGs in our data, 282 genes were classified as “cell wall”, with 66 up-regulated genes and 216 down-regulated genes (Supplement Table S7). The MapMan “cell wall” category contains eight sub-categories but only the subcategories “cell wall modification” and “cell wall pectin esterases” contained more than 50% up-regulated DEGs (Supplement Table S7). Thus, mainly genes involved in modification of cell walls were activated in drought-stressed wood.

### 2.6. Fiber Cell Wall Thickness Is Correlated with ABA Concentrations

**Figure 8:**
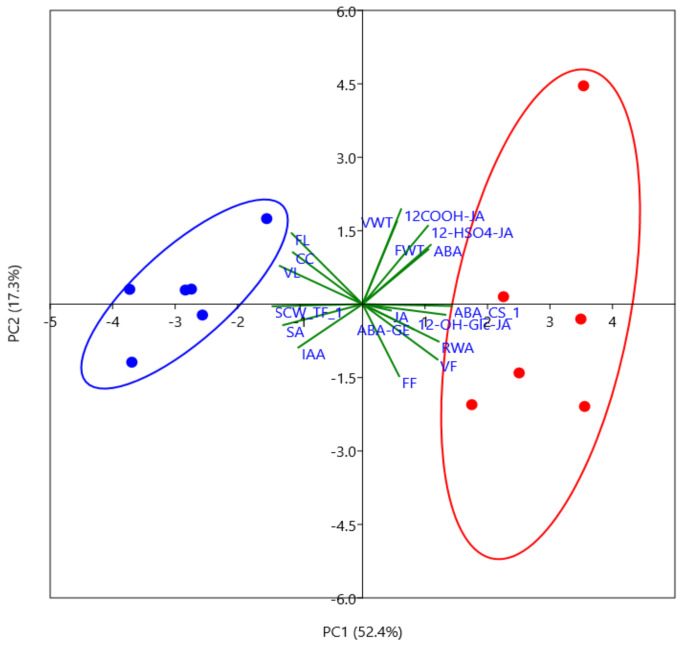
Principle component analysis (PCA) of wood anatomical traits, phytohormone concentrations and transcript abundances of genes involved in ABA signaling and the secondary cell wall (SCW) cascade in poplar (hybrid T89) wood. Red dots indicate drought-treated and blue dots well-watered samples. Abbreviations: ABA: abscisic acid, ABA-GE: ABA glucose ester, IAA: indole acetic acid, JA: jasmonic acid, SA: salicylic acid, 12HSO4-JA: 12-hydroxy jasmonoyl sulfate, 12COOH-JA: 12-hydroxy jasmonoyl carboxylate, 12-OH-Gluc-JA: 12-hydroxy jasmonoyl-1-glucose, VWT: vessel cell wall thickness, FWT: fiber cell wall thickness, VF: vessel frequency, FF: fiber frequency, VL: vessel lumen area, FL: fiber lumen area, CC: cambium cell layers and RWA: relative wall area. Original data were used for cell wall anatomical traits and phytohormones. The transcript levels of the genes constituting the ABA core signaling pathway (ABA_CS) and the transcription factors in the SCW cascade (TF_SCW) were ordinated and PC1 was used (Supplement Table S8).

To evaluate relationships of wood anatomical properties with drought-responsive phytohormones and transcriptional regulation of ABA signaling and the SCW cascade, we conducted principle component analysis (PCA) (Figure 8). To include information for transcriptional regulation, we conducted ordination of the SCW-related transcription factors and of the ABA core signaling pathway, each resulting in only one significant PC (Supplemental Table S7). We analyzed the relationship of anatomical properties, phytohormones, and the main components for PCs of the SCW cascade and ABA core signaling by PCA. Altogether, PC1 and PC2 explained 69.7% of the variation of anatomical properties (Figure 8). Drought-stressed and unstressed samples were clearly separated along PC1. The SCW-related PC correlated with unstressed and ABA core signaling with stressed wood properties (Figure 8). SCW-related PC correlated with high SA, large vessel lumina and high number cambial cell layers, indicating vigorous growth (Figure 8). High vessel frequency, high relative cell wall area, and enhanced JAs and ABA formed a group of parameters that were indicative for drought-stressed wood (Figure 8). ABA concentrations and fiber cell wall thickness were closely correlated vectors in our study (Figure 8).

## 3. Discussion

### 3.1. ABA Is Strongly Regulated in Drought-Stressed Wood

Our knowledge on the presence and functions of phytohormones in wood increased in recent years [37,73,74]. Secondary growth and xylem development are regulated by cytokinins, auxin, jasmonic acid, brassinosteroids, etc., [75]. ABA and auxin show antagonistic fluctuations in seasonal growth of trees [76,77]. ABA is instrumental for dormancy [78], but its role in the transcriptional regulation of wood formation is just emerging. Here, we demonstrate that wood contained high basal levels of ABA in non-stressed poplars and showed the most drastic increases in response to drought compared to roots or leaves. ABA tissue concentrations are controlled by metabolic and transport processes. Long-distance transport of ABA takes place in the xylem sap [79,80] and short distance from cell to cell by membrane transporters [81]. We found that the poplar homologs of ABA export proteins were upregulated and that import proteins were transcriptionally down-regulated, suggesting a shift toward ABA efflux under drought. A novel result was that transcripts for the whole suite of enzymes required for ABA biosynthesis were present in wood. *PtAAO3.3* (name according to Phytozome and PopGenIE) is more closely related to *AtAAO4* than to *AtAAO3*, which catalyzes the final step from abscisic aldehyde to ABA in *Arabidopsis* [82]. However, in *Arabidopsis aao3* mutants, ABA biosynthesis was, at least partly rescued, probably because *AAO4* or *AAO2* acted as back-up systems [82]. In the light of these results, it is likely that the AAO4 homolog PtAAO3.3 took over the oxidation of abscisic aldehyde to ABA in poplar.

Besides biosynthesis and transport, the concentration of ABA is further controlled by (i) catabolism starting with hydroxylation and conversion to phaseic acid or (ii) by conjugation with glucose and vacuolar storage [83]. In stressed *Arabidopsis* and barley seeds, phaseic acid and ABA-GE were much higher than free ABA, indicating activation of both pathways [84,85]. We observed high levels of ABA-GE in drought-stressed leaves, where it may serve as a transient store to release free ABA by hydrolysis, when needed [79,81,86]. In contrast to leaves, in wood, we found only a small increase in ABA-GE and no transcripts for homologs of two β-glucosidases *BG1* and *BG2*, which catalyze the transformation of ABA-GE to active ABA in *Arabidopsis* [87,88]. Furthermore, transcript levels of glucosyltransferase *AtUGT71B6,* which catalyzes ABA conjugation in *Arabidopsis* [89], were strongly suppressed in drought-stressed wood. Therefore, strong regulation of ABA levels by the conjugation pathway appears unlikely. Our results rather support ABA degradation in wood because the homologs of *Arabidopsis CYP707As*, genes encoding ABA 8′-hydroxylases [90], were strongly up-regulated. Altogether, our results suggest that conjugation and storage may regulate ABA levels in leaves, whereas in roots and wood other control mechanisms might be active. Based on the molecular data, it is conceivable that ABA levels in wood are governed by biosynthesis and degradation and that wood is a source rather than a sink of ABA. These speculations need to be tested urgently by functional analyses.

### 3.2. Drought Uncovers Antagonistic Effects on Wood Anatomy, Transcriptional Regulation of the SCW Cascade and ABA Core Signaling

Drought caused typical physiological changes such as decreasing stomatal conductance, indicating that the poplars exhibited a water-saving strategy [91]. Under these conditions, cambial activity was strongly diminished, consequently resulting in severely suppressed radial growth. These results are known consequences of decreased auxin levels [58]. In agreement with other studies [14,15,92,93], the poplars formed smaller but more vessels and produced thicker fiber cell walls in the secondary xylem. A novel result was that the differences between normal and drought-induced wood were accompanied by antagonistic regulation of phytohormones, SA versus jasmonates/ABA on the one hand and of the SCW cascade and the ABA core signaling pathway on the other hand. Whether SA is required for normal wood formation remains to be elucidated but it has been shown that SA promotes lignification [94]. Under biotic stress, the balance between SA and jasmonates/ABA evokes differential defense responses, which involve cell wall modifications to restrict invading pathogens [95,96]. Since cell walls are also remodeled by abiotic stress [16,97,98,99,100,101], our results might imply that antagonistic SA versus jasmonates/ABA signaling pathways were recruited for wood stress acclimation.

Cell wall thickening is an important response to enhance stability and prevent conduit collapse when the pressure on hydraulic system increases under drought [91,102]. An unexpected result in our study was that the SCW cascade was transcriptionally suppressed, despite thicker fiber walls in stressed plants. The model for the SCW cascade (as shown in Figure 6) has originally been based on genetic analyses of secondary cell wall formation in *Arabidopsis* [28] and found its equivalent in poplar [19,103]. Here, we report consistent patterns with this model since critical steps in xylem formation such as *VNDs* and their down-stream targets for programmed cell death [104] showed negative co-regulation. The suppression of *NST1* in the first level was mirrored in the 2nd level of SCW cascade by strong repression of *MYB83* and *MYB46.* MYB83 and MYB46 further regulate the third level of TFs, which, in turn, influence transcription of biosynthesis genes for secondary wall components [105]. Accordingly, cellulose synthases (*CeSAs*) together with genes involved in hemicellulose production in normal cell wall formation were consistently transcriptionally down-regulated. Similar decreases for genes required for the production of cell wall components have been reported in drought-stressed *Arabidopsis* and other plant species [106,107,108]. The response patterns to drought were less clear for lignification, because we found transcriptional activation of negative (poplar homologs to *AtMYB4* and *AtMYB7*, [32,109,110]) and positive regulators (*MYB43*, *MYB58* and *MYB63*, [111,112,113]) of lignin biosynthesis. Previous studies reported that drought does not affect or at least does not increase the lignin content but may affect the lignin composition [16,97].

To obtain further information on genes potentially recruited for drought-induced cell wall thickening, we mined our database and identified a small group of cellulose synthase like genes that were transcriptionally induced under drought. Furthermore, we uncovered a huge array of genes involved in cell wall modification (expansins, xyloglucan endotransglycosylases/hydrolases, and pectin esterases) with positive—though moderate—regulation under drought. An interesting notion is that MYB62 and MYB80, which were greatly upregulated in stressed wood of our study, shift the balance of xylose and galactose residues in hemicellulose [114] and that auxin signaling also affects the composition of hemicelluloses [115]. Thus, a picture is emerging, which suggests that drought causes a switch from normal to “stress wood” formation. Actually, drought-stressed wood shows a higher saccharification potential than that of non-stressed trees [16], which implies that cell wall remodeling must have occurred. Salt stress also influences the biochemical cell wall composition [98]. Our transcriptional studies suggest that drought recruits a dedicated set of genes for cell wall biosynthesis and remodeling. This proposition implies that changes in cell wall properties are not simply downstream consequences of up- or downregulation of the SCW but must underlie distinct control mechanisms different from that of normal wood. The analyses of cell wall components were beyond the scope of the present study but it is obvious that these analyses will shed further light on the adaptive drought responses in poplar wood.

An intriguing question is whether ABA signaling might be the regulatory hub for wood formation under drought stress. Recent studies with *Arabidopsis aba*2 mutants deficient ABA biosynthesis showed delayed fiber production and decreased transcript levels for fiber marker genes (*NST1*, *SND1*, *SND2*, *IRX3*) [49]. Activated SnRK2 in the ABA core signaling pathway can phosphorylate NST1, while suppression of NST1 and SND2, which are responsible for initiation of fiber cell wall thickening [23,24,25], results in very thin xylary cell walls in *Arabidopsis nst1/snd1* double mutants [50]. Since SnRK2 can directly activate NST1 by phosphorylation and *snrk2* as well as *aba2* mutants have thinner fiber cell walls and contain less cellulose and lignin than the wildtype Liu et al. [50] proposed that ABA regulates secondary cell wall production via the ABA core signaling pathway. According to this model, upregulation of the SCW cascade would be expected under drought, when ABA levels increase and activation of the signaling pathway occurs. In apparent contrast, drought turns down the SCW cascade in the xylem of poplars in the present study as well as in other plant species [12,106,107,108]. However, these results can be reconciled if we consider that the composition of wood is changed under stress invoking a different set of genes than those producing normal cell walls under the control of the SCW cascade. Under this premise, we may speculate that ABA signaling is required for normal wood formation, whereas stress clearly leads to a suppression of the SCW cascade and activates another program for the production and apposition of cell wall compounds. The coordination of these processes remains unclear.

## 4. Materials and Methods

### 4.1. Plant Materials and Drought Treatment

Hybrid aspen *P. tremula* × *tremuloides* (T89) were maintained and multiplied by in-vitro micro propagation according to Müller et al. [116] in 1/2 MS medium [117]. Each rooted plantlet was potted into 1.5-L pot with a 1:1 mixture of soil (Fruhstorfer Erde Type Null, Hawite Gruppe GmbH, Vechta, Germany) and sand composed of one part coarse sand (Ø 0.71–1.25 mm) and one part fine sand (Ø 0.4–0.8 mm). Plants were maintained in a greenhouse under the following conditions: air temperature: 22 °C, relative humidity: 60%, light period: 16 h light/8 h dark achieved by additional illumination with 100 μmol photons m^−2^ s^−1^. The plants were irrigated regularly with tap water before the drought treatment. Since the fourth week after potting, all plants were fertilized with Hakaphos Blue (Compo Expert, Muenster, Germany) solution once a week (1.5 g L^−1^, 50 mL per plant).

Eight weeks after potting, the plants were divided into three groups: control, moderate drought treatment, and severe drought treatment with eight biological replicates in each group. The plants were randomized among four different greenhouse chambers. Irrigation was carefully controlled during the treatment phase of 4 weeks. Soil moisture in the pot of each plant was measured with a tensiometer (HH2 Moisture Meter version 2.3, Delta-T Devices, Cambridge, UK) every day. The treatments were performed similar as described previously [118]. Control plants were well-watered exhibiting soil moistures around 0.35 m^3^ m^−3^ during the whole treatment period (Figure 1A). Moderate drought stress was gradually initiated by lowering the soil moisture of drought-treated plants reaching 0.15 m^3^ m^−3^ in the third week and thereafter kept between 0.10 and 0.15 m^3^ m^−3^ for one further week (Figure 1A). Severe drought treatment was achieved by stopping irrigation until the plants wilted. The wilting threshold was reached when soil moisture decreased to 0.10 m^3^ m^−3^, and then a small amount of water (~5 to 10 mL per day) was added per pot per day to keep the plants alive (with soil moisture around 0.10 m^3^ m^−3^) until the end of the experiment (Figure 1A).

Plant height and basal stem diameter were measured once a week. Stomatal conductance was measured using a porometer (AP4, Delta-T Devices, Cambridge, UK) on the first fully developed leaf from the top once a week. Leaves formed during the treatment period of each plant were counted each week.

### 4.2. Sampling and Biomass

At the harvest date, the plants were cut at the stem bottom and the fresh weight of all leaves and the stem were determined. Two to three fully developed leaves were collected separately from each plant, and were shock-frozen in liquid nitrogen and then were stored at −80 °C. Pieces of the basal stem (right above soil, about 3 cm long) of each plant were fixed in FAE solution (2% formaldehyde, 5% acetic acid and 63% ethanol) for anatomical analysis. Further stem pieces were debarked. The debarked wood was immediately frozen in liquid nitrogen and then stored at −80 °C.

Three fresh leaves from top, middle, and lower positions at the stem of each plant were collected, weighed, scanned to determine the leaf area (Image J, https://imagej.net/ImageJ (accessed on 23 January 2018), [119]) and then dried in an oven at 60 °C for one week. The measurements were used to determine the specific leaf area (SLA, cm g^−1^, Equation (1)), area per leaf (cm^2^ leaf^−1^, Equation (2)) and whole plant leaf area (cm^2^ plant^−1^, Equation (3)).

(1) $$SLA\;({\mathrm{cm}}^{2}\;g^{-1})=\frac{\mathrm{Leaf}\;\mathrm{area}\;\mathrm{of}\;\mathrm{the}\;\mathrm{scanned}\;\mathrm{leaves}\;({\mathrm{cm}}^{2})}{\mathrm{dry}\;\mathrm{weight}\;\mathrm{of}\;\mathrm{the}\;\mathrm{scanned}\;\mathrm{leaves}\;\left(g\right)}$$

(2) $$\mathrm{Area}\;\mathrm{per}\;\mathrm{leaf}\;({\mathrm{cm}}^{2})=\frac{\mathrm{Leaf}\;\mathrm{area}\;\mathrm{of}\;\mathrm{the}\;\mathrm{scanned}\;\mathrm{leaves}\;({\mathrm{cm}}^{2})}{\mathrm{Number}\;\mathrm{of}\;\mathrm{the}\;\mathrm{scanned}\;\mathrm{leaves}\;\left(N\right)}$$

(3) $$\mathrm{Whole}\;\mathrm{plant}\;\mathrm{leaf}\;\mathrm{area}\;\left({\mathrm{cm}}^{2}\;{\mathrm{plant}}^{-1}\right)=\frac{\mathrm{Dry}\;\mathrm{weight}\;\mathrm{of}\;\mathrm{all}\;\mathrm{leaves}\;\mathrm{of}\;\mathrm{the}\;\mathrm{plant}\;\left(g\right)\times \mathrm{Leaf}\;\mathrm{area}\;\mathrm{of}\;\mathrm{the}\;\mathrm{scanned}\;\mathrm{leaves}\;\left({\mathrm{cm}}^{2}\right)}{\mathrm{Dry}\;\mathrm{weight}\;\mathrm{of}\;\mathrm{the}\;\mathrm{scanned}\;\mathrm{leaves}\;\left(g\right)}$$

A fully developed top leaf was collected from each plant to determine the leaf relative water content (%) of each plant as described by Bogeat-Triboulot et al. [15].

The roots were removed from the pots, quickly washed with tap water, surface-dried between tissue paper, and weighed. Aliquots of root tips were immediately frozen in liquid nitrogen and stored at −80 °C. Aliquots of each tissue (leaves, stem and roots) were weighed, dried in an oven at 60 °C for one week, and used to determine the dry weight. Tissue biomass was determined according to Equation (4). Plant biomass was calculated as the sum of biomass of the tissues: leaf, stem, and root.

(4) $$\mathrm{Whole}\;\mathrm{tissue}\;\mathrm{biomass}\;\left(g\right)=\frac{\mathrm{Dry}\;\mathrm{weight}\;\mathrm{of}\;\mathrm{the}\;\mathrm{aliquot}\;\left(g\right)\times \mathrm{Total}\;\mathrm{fresh}\;\mathrm{weight}\;\mathrm{of}\;\mathrm{the}\;\mathrm{tissue}\;\left(g\right)}{\mathrm{Fresh}\;\mathrm{weight}\;\mathrm{of}\;\mathrm{the}\;\mathrm{aliquot}\;\left(g\right)}\;$$

### 4.3. Wood Anatomical Analysis

Five biological replicates of control and drought-stressed plants were prepared for wood anatomical analysis. The preparation of stem cross sections and the analyses of wood anatomical traits, such as vessel density, vessel lumen area, vessel cell wall thickness, fiber density, fiber lumen area, fiber cell wall thickness, and the percentage of cell wall area, were conducted as described by Wildhagen et al. [16]. The analysis of wood traits was conducted in the outer layer of the xylem next to the cambium, to include only wood that was formed during the four-week stress phase.

### 4.4. Phytohormone Measurements

Frozen samples of leaves, wood, and root tips (*n* = 5 per tissue per treatment) were milled in cooled vessels in a ball mill (MM400, Retsch, Haan, Germany) to a fine powder keeping the sample frozen. From each sample, frozen milled materials (100 mg) were extracted with 0.75 mL of methanol containing 10 ng D_4_-SA, 10 ng D_6_-ABA, 10 ng D_5_-JA (all three from C/D/N Isotopes Inc., Pointe-Claire, Canada), 20 ng D_5_-IAA (Eurisotop, Freising, Germany), as internal standards. Phytohormones were extracted with methyl-*tert*-butyl ether (MTBE), reversed phase-separated using an ACQUITY UPLC^®^ system (Waters Corp., Milford, MA, USA) and analyzed by nanoelectrospray (nanoESI) (TriVersa Nanomate^®^; Advion BioSciences, Ithaca, NY, USA) coupled with an AB Sciex 4000 QTRAP^®^ tandem mass spectrometer (AB Sciex, Framingham, MA, USA) employed in scheduled multiple reaction monitoring mode [120]. The mass transitions are shown in Supplement Table S9.

### 4.5. Statistical Analyses of Physiological Data

The software programs R 3.4.2 [121] and Origin Pro 8.5G (OriginLab, Northampton, MA, USA) were used for statistical analyses and figure generation. Data of plant height, stem diameter, biomass of each tissue, gas exchange, anatomical traits and hormone concentrations were analyzed. One-way ANOVA was applied to compare the means between different treatments. Normality and homogeneity of variances were assessed visually by plotting residuals. Logarithmic (log2) transformation was applied to achieve normal distribution if necessary. When *p*-value < 0.05, Tukey test was applied as post-hoc for pairwise analysis.

### 4.6. RNA Extraction and Sequencing

RNA was extracted from wood using six biological replicates per treatment. Frozen samples were milled using a ball mixer mill (MM400, Retsch, Haan, Germany). About 160 to 200 mg material was used for RNA extraction using a modified CTAB protocol [122]. Quality and concentration of the total RNA was measured by an Agilent 2100 Bioanalyzer RNA Nano assay (Agilent Technologies, Santa Clara, CA, USA). Total RNA samples (with RIN > 6.5) were diluted to 40 ng/µL with nucleotide free water, and 100 µL of each sample was used for library preparation using the “TruSeq mRNA Sample Prep kit v2” (Illumina, San Diego, CA, USA). Final libraries were quantified using the Qubit 2.0 Fluorometer (Invitrogen, Carlsbad, CA, USA) and quality tested by Agilent 2100 Bioanalyzer High Sensitivity or DNA 1000 assay (Agilent Technologies). Libraries were loaded on Illumina cBot for cluster generation on the flow cell, and sequenced in 50 bp single-end mode at six-fold multiplex on the Illumina HiSeq2000 (Illumina). Raw data were processed using the CASAVA 1.8.2 version of the Illumina pipeline for both format conversion and de-multiplexing. All RNA-seq data have been deposited in the ArrayExpress database at EMBL-EBI (www.ebi.ac.uk/arrayexpress (accessed on 10 January 2019)) under accession number E-MTAB-7589.

### 4.7. Bioinformatic Analyses

The raw data of each sample consisted of ~21 to 35 million reads. Processing of the raw data was performed with the FASTX toolkit (http://hannonlab.cshl.edu/fastx_toolkit/ (accessed on 3 May 2018)). Using FASTQ Trimmer, from the ends of the reads, all nucleotides with a Phred quality score below 20 were removed. Then, the sequences smaller than 25 bp or with a Phred quality score below 20 for 10% of the nucleotides were discarded. The FASTQ Clipper (http://hannonlab.cshl.edu/fastx_toolkit/ (accessed on 4 May 2018)) was used to remove adapter sequences and primers. After processing, ~10 to 18 million reads per sample remained.

The processed sequences were mapped against the *P. trichocarpa* transcriptome v3.1 [123] using Bowtie 2 [124]. Bowtie mapping files were summarized to transcript count tables in R. To find transcripts with significantly increased or decreased abundance, the DEseq2 package [125] implemented in R was used. GO term enrichment analyses were conducted using Ontologizer [126] with term-for-term approach and Benjamini-Hochberg corrections. Further GO analyses were conducted with Metascape [127] and Mapman binning with the Classification Superviewer (http://bar.utoronto.ca/ (accessed on 15 October 2018)) using the best *Arabidopsis* match of the poplar DEGs. Cluster analyses were conducted with ClustVis and standard settings [72].

## Figures and Tables

**Table 1:** Leaf area and biomass of poplar (hybrid T89) in response to moderate and severe drought stress and well-watered conditions. Data show means (SE) of *n* = 8. Different letters indicate significant differences of means at *p* ≤ 0.05, (one-way ANOVA, Tukey post-hoc test). SLA = specific leaf area.

Treatments	Well-Watered	Moderate Drought	Severe Drought	*p*-Value
Leaf number ^#^	15.38 (0.53) c	11.50 (0.33) b	7.50 (0.33) a	**<0.001**
Leaf size (cm^2^ leaf^−1^)	57.24 (2.28) b	51.96 (2.35) b	43.36 (2.40) a	**0.002**
SLA (cm^2^ g^−1^ dry mass)	306.7 (7.6) a	313.5 (12.7) a	303.0 (11.0) a	0.779
Whole-plant leaf area (cm^2^ plant^−1^)	1251.4 (55.4) c	819.5 (20.4) b	478.8 (32. 9) a	**<0.001**
Biomass of leaves (g plant^−1^)	4.18 (0.16) c	2.77 (0.11) b	2.11 (0.17) a	**<0.001**
Biomass of stem (g plant^−1^)	2.68 (0.23) c	1.65 (0.13) b	1.06 (0.07) a	**<0.001**
Biomass of roots (g plant^−1^)	1.86 (0.19) a	1.45 (0.16) a	1.40 (0.09) a	0.099
Whole-plant biomass (g plant^−1^)	8.71 (0.53) b	5.88 (0.38) a	4.56 (0.28) a	**<0.001**
Root-to-shoot ratio	0.70 (0.05) a	0.87 (0.06) a	1.34 (0.10) b	**<0.001**
Relative leaf water content (%)	92.26 (2.77) b	82.76 (2.99) ab	74.45 (3.68) a	**0.003**

^#^ Leaf number indicates the number of leaves formed during the treatment period per plant.

**Table 2:** Concentrations of phytohormones in leaves, wood, and fine roots of poplar (hybrid T89) in response to drought treatments. Data show means (SE) of *n* = 3 to 5. Two-way ANOVA was applied to test the difference between tissues and treatments. *p*-values of comparison between treatments (treat), between leaves, wood, and roots (tissue) and the interaction effect of tissues and treatments (treat:tissue) are listed in the last column. Tukey-test was applied post-hoc for each tissue separately and means that differ at *p* ≤ 0.05 are indicated by different letters. fw refers to fresh weight.

Phytohormone (nmol g^−1^ fw)	Tissue	Well-Watered	Moderate Drought	Severe Drought	*p*-Values	
SA	Leaf	4.81 (0.89) a	5.09 (1.27) a	3.88 (1.29) a	treat	**0.032**
	Root	0.74 (0.15) a	4.64 (0.63) c	2.49 (0.18) b	tissue	**<0.001**
	Wood	0.72 (0.05) b	0.50 (0.08) a	0.42 (0.08) a	treat:tissue	**0.020**
IAA	Leaf	NA	NA	NA	treat	0.310
	Root	NA	NA	NA	tissue	NA
	Wood	0.44 (0.05) a	0.39 (0.06) a	0.32 (0.06) a	treat:tissue	NA
JA	Leaf	0.12 (0.05) a	0.43 (0.31) a	0.21 (0.10) a	treat	0.206
	Root	0.07 (0.02) a	0.15 (0.08) a	0.08 (0.01) a	tissue	0.290
	Wood	0.07 (0.02) a	0.21 (0.11) a	0.20 (0.15) a	treat:tissue	0.845
12-HSO_4_-JA ^#^	Leaf	78.79 (57.87) a	47.04 (34.56) a	52.21 (33.24) a	treat	0.826
	Root	0.01 (0.005) a	0.03 (0.02) a	0.02 (0.01) a	tissue	**0.003**
	Wood	0.005 (0.001) a	0.006 (0.003) a	0.008 (0.002) a	treat:tissue	0.941
12-OH-Glc-JA ^#^	Leaf	352.48 (252.74) a	187.42 (128.69) a	216.83 (150.81) a	treat	0.759
	Root	0.24 (0.10) a	0.98 (0.60) a	0.91 (0.35) a	tissue	**0.003**
	Wood	0.13 (0.03) a	0.26 (0.09) a	0.74 (0.16) b	treat:tissue	0.887
12-COOH-JA ^#^	Leaf	138.86 (72.63) a	114.28 (93.76) a	94.89 (73.91) a	treat	0.914
	Root	0.66 (0.79) a	0.32 (1.4) a	0.52 (1.30) a	tissue	**0.002**
	Wood	1.31 (0.30) a	1.4 (0.61) a	1.56 (0.36) a	treat:tissue	0.983
ABA	Leaf	0.37 (0.04) a	3.95 (2.52) b	4.94 (1.76) b	treat	**0.008**
	Root	nd a	0.31 (0.09) b	0.38 (0.08) b	tissue	**0.013**
	Wood	0.42 (0.05) a	3.00 (1.89) ab	6.09 (2.48) b	treat:tissue	0.303
ABA-GE ^$^	Leaf	nd a	4.34 (1.77) b	7.28 (1.74) b	treat	**0.021**
	Root	0.15 (0.05) a	1.04 (0.56) a	0.61 (0.12) a	tissue	**<0.001**
	Wood	0.30 (0.09) a	0.57 (0.08) ab	0.68 (0.18) b	treat:tissue	**0.002**

^#^ The concentrations of JAs are shown as relative values based on the pure standard of JA. ^$^ data were log-transformed for ANOVA analysis. SA: salicylic acid, IAA: indole acetic acid, JA: jasmonic acid, 12-HSO_4_-JA: 12-hydroxy jasmonoyl sulfate, 12-OH-Gluc-JA: 12-hydroxy jasmonoyl-1-glucose, 12-COOH-JA: 12-hydroxy jasmonoyl carboxylate, ABA: abscisic acid, ABA-GE: ABA glucose ester, nd: below the detection limit, 0 was used for calculation, NA: not available.

**Table 3:** Anatomical characteristics of poplar (hybrid T89) in response to severe drought treatment. Data show means (SE) of *n* = 5. One-way ANOVA was conducted to compare the differences among treatments. *p*-values for mean that differ at *p* < 0.05 are indicated by fat letters.

Anatomical Traits	Control	Drought	*p*-Value
Vessel frequency (number of vessels mm^−2^)	240.20 (11.58)	503.97 (46.74)	**<0.001**
Vessel lumen size (μm^2^)	749.19 (39.57)	339.86 (37.95)	**<0.001**
Fiber frequency (number of fibers mm^−2^)	4162.0 (113.8)	4865.0 (646.5)	0.310
Fiber lumen size (μm^2^)	86.09 (3.35)	56.36 (7.02)	**0.003**
Vessel cell wall thickness (µm) Double fiber cell wall thickness (μm)	1.28 (0.09) 3.79 (0.13)	1.45 (0.12) 4.22 (0.11)	0.268 **0.026**
Fraction of cell wall area (%)	39.26 (1.70)	48.40 (1.77)	**<0.001**
Total cell wall area in mature xylem formed under treatments (mm^2^)	2.35 (0.18)	0.99 (0.10)	**<0.001**
Number of cambial cell layers	6.6 (0.5)	3.8 (0.3)	**<0.001**

## Data Availability

RNA-seq data are available under accession number E-MTAB-7589 in the ArrayExpress database at EMBL-EBI (www.ebi.ac.uk/arrayexpress (accessed on 15 July 2021)). Other data are available in the Supplementary Materials.

## Supplementary Materials

The following are available online at https://www.mdpi.com/article/10.3390/ijms22189899/s1. Table S1: Transcript abundances and statistical information of genes in poplar wood in response to severe drought treatment. Table S2: GO-terms for DEGs that were enriched in poplar wood (hybrid T89) in response to severe drought treatment. Table S3: DEGs involved in ABA biosynthesis, signaling, and homeostasis in poplar wood (hybrid T89) in response to severe drought stress. Table S4: DEGs involved in IAA and JA biosynthesis pathways. Table S5: Differentially expressed transcription factors governing secondary cell wall (SCW) formation in poplar wood (hybrid T89) in response to severe drought stress. Table S6: DEGs involved in lignin biosynthesis in poplar wood (hybrid T89) in response to severe drought stress. Table S7: DEGs that were classified by MapMan in the category “cell wall” in poplar wood (hybrid T89). Table S8: Results of the principle component analyses (PCA) for ABA core signaling (ABA_CS) and transcription factors regulating the secondary cell wall formation cascade (SCW_TF). Table S9: Mass transitions and corresponding conditions for the identification of phytohormones shown in Table 2. Figure S1: GO terms enriched with up-(A) and down-(B) regulated genes in poplar wood (hybrid T89) in response to severe drought treatment. Figure S2: Transcriptional regulation of genes involved in IAA (A) and JA (B) biosynthesis pathways. Figure S3: Hierarchical cluster analysis of genes in poplar wood (hybrid T89) related to hemicellulose formation.
