# Supplementary material for: Wood Formation under Severe Drought Invokes Adjustment of the Hormonal and Transcriptional Landscape in Poplar
Source: Int J Mol Sci. 2021 Sep 13;22(18):9899. doi: 10.3390/ijms22189899 (PMC8493802; doi:10.3390/ijms22189899)
Supplement: Supplementary file 1 [file ijms-22-09899-s001.zip › Supplementary Materials_20210913.pdf]

Supplement Table S1 Transcript abundances and statistical information of genes in poplar wood in response to severe drought treatment.

Xylem of hybrid poplar (T89) was harvested after 4 weeks of drought and used for RNA sequencing. The data show Potri IDs, names of Arabidopsis orthologs, log2-fold changes of drought /control (log2fc.t89.c.d), Bonferroni-adjusted p-values of the comparison drought/control (padj.t89.c.d) and rarefied count data for each sample (tdr). Control: tdr_1 , tdr_2, tdr_4, tdr_6, tdr_9, tdr_12, drought: tdr_3, tdr_5, tdr_7, tdr_8, tdr_10, tdr_11

The table has been uploaded as a separate excel-file.

Supplement Table S2 GO-terms for DEGs that were enriched in poplar wood (hybrid T89) in response to severe drought treatment

P values were adjusted using Benjamini-Hochberg correction.

ID	Num of parents	P value adjusted	name
**GO:0044445**	2	2.15E-54	cytosolic part
**GO:0005840**	3	1.22E-49	ribosome
**GO:0044391**	4	7.30E-49	ribosomal subunit
**GO:0030529**	2	1.32E-41	ribonucleoprotein complex
**GO:0005198**	1	1.93E-39	structural molecule activity
**GO:0043232**	2	2.75E-34	intracellular non-membrane-bounded organelle
**GO:0043228**	1	2.75E-34	non-membrane-bounded organelle
**GO:0006412**	3	9.38E-26	translation
**GO:0001510**	2	1.04E-21	RNA methylation
**GO:0005829**	1	6.50E-15	cytosol
**GO:0022626**	2	3.21E-12	cytosolic ribosome
**GO:0032991**	1	6.05E-10	macromolecular complex
**GO:0044085**	1	1.59E-08	cellular component biogenesis
**GO:0003674**	1	1.65E-08	molecular_function
**GO:0003735**	1	6.17E-08	structural constituent of ribosome
**GO:0071554**	1	9.22E-08	cell wall organization or biogenesis
**GO:0009414**	2	1.24E-05	response to water deprivation
**GO:0034470**	2	2.05E-05	ncRNA processing
**GO:0009832**	2	2.66E-05	plant-type cell wall biogenesis
**GO:0003774**	1	2.66E-05	motor activity
**GO:0009451**	2	3.91E-05	RNA modification
**GO:0009699**	4	4.51E-05	phenylpropanoid biosynthetic process
**GO:0044446**	3	2.66E-04	intracellular organelle part
**GO:0006970**	2	3.77E-04	response to osmotic stress
**GO:0008092**	1	4.09E-04	cytoskeletal protein binding
**GO:0042221**	1	4.09E-04	response to chemical
**GO:0009611**	1	6.17E-04	response to wounding
**GO:0032403**	1	6.40E-04	protein complex binding
**GO:0003700**	3	0.001175	sequence-specific DNA binding transcription factor activity
**GO:0008152**	1	0.001606	metabolic process
**GO:0022613**	1	0.001764	ribonucleoprotein complex biogenesis
**GO:0009804**	2	0.002444	coumarin metabolic process
**GO:0009415**	3	0.002444	response to water
**GO:0009805**	3	0.002664	coumarin biosynthetic process
**GO:0046906**	2	0.002989	tetrapyrrole binding
**GO:0044422**	2	0.004171	organelle part
**GO:0005886**	3	0.004556	plasma membrane
**GO:0009538**	4	0.004565	photosystem I reaction center

**GO:0010583**	1	0.005874	response to cyclopentenone
**GO:0009698**	4	0.006779	phenylpropanoid metabolic process
**GO:0071944**	1	0.009255	cell periphery
**GO:0006090**	1	0.009657	pyruvate metabolic process
**GO:2000652**	3	0.009755	regulation of secondary cell wall biogenesis
**GO:0044444**	2	0.00998	cytoplasmic part
**GO:0048878**	1	0.013855	chemical homeostasis
**GO:0042546**	2	0.013855	cell wall biogenesis
**GO:0009719**	1	0.014786	response to endogenous stimulus
**GO:0009407**	3	0.014911	toxin catabolic process
**GO:0006720**	1	0.014911	isoprenoid metabolic process
**GO:0019748**	1	0.015557	secondary metabolic process
**GO:0071669**	1	0.02061	plant-type cell wall organization or biogenesis
**GO:0009267**	3	0.024886	cellular response to starvation
**GO:0043414**	3	0.027311	macromolecule methylation
**GO:0032879**	2	0.029679	regulation of localization
**GO:0010817**	1	0.029679	regulation of hormone levels
**GO:0019363**	4	0.030698	pyridine nucleotide biosynthetic process
**GO:0016301**	2	0.035103	kinase activity
**GO:0004364**	1	0.038806	glutathione transferase activity
**GO:0031984**	2	0.043544	organelle subcompartment
**GO:0009521**	4	0.046414	photosystem
**GO:0032886**	2	0.046414	regulation of microtubule-based process
**GO:1901293**	4	0.046481	nucleoside phosphate biosynthetic process
**GO:0007166**	1	0.046481	cell surface receptor signaling pathway

Supplement Table S3 DEGs involved in ABA biosynthesis, signaling and homeostasis in poplar wood (hybrid T89) in response to severe drought stress

Log_2-fold changes (FC) of up-regulated genes are shown in dark red and log_2-FC of down-regulated genes in dark blue. DEGs are genes with p_{adj} (Bonferroni correction) < 0.05.

Function	Potri.ID	AGI	At.name	log_2-FC	p_{adj}
ABA synthesis	Potri.004G191300	AT3G43600	AAO2	**-0.971**	<0.001
ABA synthesis	Potri.007G044300	AT5G67030	ABA1	**2.190**	<0.001
ABA synthesis	Potri.016G073700	AT1G52340	ABA2	**-1.770**	<0.001
ABA synthesis	Potri.006G206500	AT1G52340	ABA2	**2.350**	<0.001
ABA synthesis	Potri.011G112400	AT3G14440	NCED3	**4.101**	<0.001
ABA synthesis	Potri.001G393800	AT3G14440	NCED3	**3.882**	<0.001
ABA signaling_PP2C_A	Potri.006G164600	AT4G26080	ABI1	**2.471**	<0.001
ABA signaling_PP2C_A	Potri.015G133900	AT5G51760	AHG1	**6.499**	<0.001
ABA signaling_PP2C_A	Potri.012G131800	AT5G51760	AHG1	**3.793**	<0.001
ABA signaling_PP2C_A	Potri.018G060300	AT1G72770	HAB1	**-0.700**	<0.001
ABA signaling_PP2C_A	Potri.001G245200	AT1G07430	HAI2	**6.094**	<0.001
ABA signaling_PP2C_A	Potri.009G037300	AT1G07430	HAI2	**6.911**	<0.001
ABA signaling_PP2C_A	Potri.012G002700	AT2G29380	HAI3	**8.159**	<0.001
ABA signaling_PP2C_A	Potri.001G092100	AT2G29380	HAI3	**8.678**	<0.001
ABA signaling_PP2C_A	Potri.015G018800	AT2G29380	HAI3	**7.139**	<0.001
ABA signaling_PP2C_A	Potri.008G059200	AT3G11410	PP2CA	**2.608**	<0.001
ABA signaling_PP2C_A	Potri.010G199600	AT3G11410	PP2CA	**1.850**	<0.001
ABA signaling_ RCAR	Potri.014G097100	AT1G01360	RCAR1/PYL9	**2.487**	<0.001
ABA signaling_ RCAR	Potri.012G000800	AT5G53160	RCAR3/PYL8	**-0.819**	<0.001
ABA signaling_ RCAR	Potri.001G092500	AT5G53160	RCAR3/PYL8	**0.847**	<0.001
ABA signaling_ RCAR	Potri.008G073400	AT2G40330	RCAR9/PYL6	**-1.074**	0.049
ABA signaling_ RCAR	Potri.010G183900	AT2G40330	RCAR9/PYL6	**-2.856**	<0.001
ABA signaling_ RCAR	Potri.016G125400	AT2G38310	RCAR10/PYL4	**-3.226**	<0.001
ABA signaling_ RCAR	Potri.003G091700	AT4G17870	RCAR11/PYL1	**-1.180**	0.047
ABA signaling_ RCAR	Potri.001G142500	AT4G17870	RCAR11/PYL1	**-2.547**	<0.001
ABA signaling_SnRK2	Potri.003G084100	AT4G33950	OST1/SnRK2.6	**-0.743**	0.017
ABA signaling_SnRK2	Potri.009G106900	AT4G33950	OST1/SnRK2.6	**0.578**	<0.001
ABA downstream_TF	Potri.009G101232	AT4G34000	ABF3	**1.879**	<0.001
ABA downstream_TF	Potri.004G140600	AT4G34000	ABF3	**1.810**	<0.001
ABA downstream_TF	Potri.009G101166	AT4G34000	ABF3	**2.099**	<0.001
Encode ABA hydroxylases	Potri.004G235400	AT4G19230	CYP707A1	**5.448**	<0.001
Encode ABA hydroxylases	Potri.001G242600	AT2G29090	CYP707A2	**6.901**	<0.001
Encode ABA hydroxylases	Potri.009G033900	AT2G29090	CYP707A2	**3.896**	<0.001
Encode ABA hydroxylases	Potri.002G126100	AT3G19270	CYP707A4	**1.547**	0.002
Encode ABA hydroxylases	Potri.014G029100	AT3G19270	CYP707A4	**5.172**	<0.001
Encode ABA hydroxylases	Potri.004G140900	AT3G19270	CYP707A4	**4.725**	<0.001
Encode ABA hydroxylases	Potri.009G101700	AT3G19270	CYP707A4	**2.824**	<0.001
Convert ABA to ABA-GE	Potri.016G017300	AT3G21780	UGT71B6	**-1.852**	<0.001

transporters	Potri.017G051300	AT1G17840	ABCG11	**-4.506**	<0.001
transporters	Potri.005G073100	AT1G17840	ABCG11	**-1.454**	<0.001
transporters	Potri.004G236500	AT1G31770	ABCG14	**-2.316**	0.010
transporters	Potri.003G046800	AT1G31770	ABCG14	**-1.790**	0.019
transporters	Potri.010G153700	AT3G53480	ABCG37	**-2.447**	<0.001
transporters	Potri.010G153600	AT3G53480	ABCG37	**-1.988**	<0.001
transporters	Potri.006G115000	AT1G66950	ABCG39	**-1.177**	<0.001
transporters_ABA export	Potri.019G083000	AT1G71960	ABCG25	**1.194**	0.012
transporters_ABA import	Potri.001G175700	AT1G15520	ABCG40	**-1.122**	0.028
transporters_ABA import	Potri.003G057301	AT1G15520	ABCG40	**-1.084**	0.039
transporters_ABA import	Potri.003G183200	AT1G15520	ABCG40	**-1.400**	0.041
transporters_ABA import	Potri.003G057351	AT1G15520	ABCG40	**-2.701**	<0.001
transporters_ABA import	Potri.006G248500	AT1G15520	ABCG40	**-3.002**	<0.001
transporters_ABA import	Potri.018G032900	AT1G15520	ABCG40	**-2.768**	<0.001
transporters_ABA import	Potri.001G048900	AT1G15520	ABCG40	**-1.605**	<0.001
transporters_ABA import	Potri.012G070700	AT1G69850	AIT1/NRT1.2	**-1.057**	0.019
transporters_ABA import	Potri.010G034300	AT1G69850	AIT1/NRT1.2	**0.747**	0.044

Supplement Table S4 DEGs involved in IAA and JA biosynthesis pathways

Log_2-fold changes (FC) of up-regulated genes are shown in dark red and log_2-FC of down-regulated genes in dark blue. DEGs are genes with p_{adj} (Bonferroni correction) < 0.05.

Function	Potri.ID	AGI	At.name	log_2-FC	p_{adj}
IAA biosynthesis	Potri.005G237300	AT1G19920	ASA1	**-0.574**	**0.048**
IAA biosynthesis	Potri.009G044300	AT2G29690	ASA2	**0.591**	**0.002**
IAA biosynthesis	Potri.012G082700	AT5G17990	PAT1	**-0.982**	**0.001**
IAA biosynthesis	Potri.008G069500	AT1G07780	PAI1	**1.896**	**0.004**
IAA biosynthesis	Potri.005G217700	AT3G54640	TSA1	**-0.644**	**<0.001**
IAA biosynthesis	Potri.010G044500	AT1G70560	TAA1	**2.38**	**<0.001**
IAA biosynthesis	Potri.006G098300	AT2G38120	AUX1	**-2.336**	**<0.001**
IAA biosynthesis	Potri.016G113600	AT2G38120	AUX1	**-1.897**	**<0.001**
IAA biosynthesis	Potri.006G243400	AT5G25620	YUC6	**-2.929**	**<0.001**
IAA biosynthesis	Potri.018G036800	AT5G25620	YUC6	**-2.403**	**<0.001**
IAA biosynthesis	Potri.002G045000	AT5G43700	IAA4	**-1.22**	**0.048**
IAA biosynthesis	Potri.005G053800	AT5G43700	IAA4	**-2.241**	**<0.001**
IAA biosynthesis	Potri.013G041300	AT5G43700	IAA4	**-2.172**	**<0.001**
IAA biosynthesis	Potri.013G024100	AT1G08980	AMI1	**0.961**	**<0.001**
IAA biosynthesis	Potri.013G024200	AT1G08980	AMI1	**1.482**	**<0.001**
JA biosynthesis	Potri.001G252900	AT5G05580	FAD8	**2.992**	**<0.001**
JA biosynthesis	Potri.010G187800	AT5G05580	FAD8	**2.591**	**0.001**
JA biosynthesis	Potri.018G132100	AT2G06925	PLA2-α	**-1.065**	**0.017**
JA biosynthesis	Potri.003G067600	AT1G17420	LOX3	**-0.698**	**0.044**
JA biosynthesis	Potri.014G177200	AT3G22400	LOX5	**-0.873**	**0.001**
JA biosynthesis	Potri.001G227100	AT3G45140	LOX2	**-1.963**	**<0.001**
JA biosynthesis	Potri.004G149000	AT5G42650	AOS	**2.401**	**<0.001**
JA biosynthesis	Potri.009G109700	AT5G42650	AOS	**-2.687**	**<0.001**
JA biosynthesis	Potri.004G102500	AT3G25780	AOC3	**1.638**	**0.002**
JA biosynthesis	Potri.008G117300	AT3G25780	AOC3	**2.49**	**<0.001**
JA biosynthesis	Potri.018G065600	AT2G06050	OPR3	**1.794**	**0.014**
JA biosynthesis	Potri.004G212100	AT2G06050	OPR3	**0.603**	**0.036**
JA biosynthesis	Potri.017G034901	AT1G20510	OPCL1	**-2.221**	**<0.001**
JA biosynthesis	Potri.017G035100	AT1G20510	OPCL1	**-2.439**	**<0.001**
JA biosynthesis	Potri.008G220400	AT3G06860	MFP2	**-1.032**	**0.002**
JA biosynthesis	Potri.010G011900	AT3G06860	MFP2	**1.019**	**0.001**
JA biosynthesis	Potri.001G051800	AT2G33150	KAT2	**0.574**	**0.014**
JA biosynthesis	Potri.001G051900	AT2G33150	KAT2	**1.043**	**0.001**
JA biosynthesis	Potri.002G216400	AT2G33150	KAT2	**-1.779**	**<0.001**
JA biosynthesis	Potri.017G103700	AT2G33150	KAT2	**1.907**	**<0.001**

Supplement Table S5 Differentially expressed transcription factors governing secondary cell wall (SCW) formation in poplar wood (hybrid T89) in response to severe drought stress

Log_2-fold changes (FC) of up-regulated genes are shown in dark red and log_2-FC of down-regulated genes in dark blue. DEGs are genes with p_{adj} (Bonferroni correction) < 0.05.

Function	Potri.ID	AGI	At.name	log_2-FC	p_{adj}
**Fine-tuning factors**	Potri.001G188800	AT1G52150	HB15	**-1.034**	<0.001
	Potri.003G050100	AT1G52150	HB15	**-0.744**	0.004
	Potri.003G166500	AT5G13180	VNI2	**-0.579**	0.005
	Potri.017G063300	AT5G13180	VNI2	**2.813**	<0.001
	Potri.001G325100	AT5G13180	VNI2	**2.816**	<0.001
	Potri.008G011300	AT3G20770	EIN3	**0.990**	0.003
**Master regulators (NAC)**	Potri.011G153300	AT2G46770	NST1	**-4.044**	<0.001
	Potri.001G448400	AT2G46770	NST1	**-3.367**	<0.001
	Potri.002G178700	AT2G46770	NST1	**-2.516**	<0.001
	Potri.007G014400	AT2G18060	VND1	**-3.388**	<0.001
	Potri.005G116800	AT2G18060	VND1	**-3.023**	<0.001
	Potri.012G126500	AT1G12260	VND4	**-2.956**	<0.001
	Potri.003G113000	AT1G12260	VND4	**-2.513**	<0.001
	Potri.015G127400	AT1G12260	VND4	**-2.425**	<0.001
	Potri.001G120000	AT1G12260	VND4	**-0.871**	0.007
**Second Level Regulators (MYB)**	Potri.001G258700	AT5G12870	MYB46	**-2.475**	<0.001
	Potri.009G053900	AT5G12870	MYB46	**-2.222**	<0.001
	Potri.009G061500	AT3G08500	MYB83	**-3.086**	<0.001
	Potri.001G267300	AT3G08500	MYB83	**-2.979**	<0.001
**Third Level Regulators**	Potri.007G135300	AT4G28500	SND2	**-3.900**	<0.001
	Potri.017G016700	AT4G28500	SND2	**-3.487**	<0.001
	Potri.011G058400	AT4G28500	SND2	**-2.581**	<0.001
	Potri.004G049300	AT4G28500	SND2	**-1.047**	0.005
	Potri.003G132000	AT1G63910	MYB103	**-4.397**	<0.001
	Potri.001G099800	AT1G63910	MYB103	**-3.286**	<0.001
	Potri.003G114100	AT4G12350	MYB42	**-2.506**	<0.001
	Potri.001G118800	AT4G12350	MYB42	**-1.756**	<0.001
	Potri.012G127700	AT4G12350	MYB42	**-1.135**	0.001
	Potri.017G130300	AT5G16600	MYB43	**1.673**	<0.001
	Potri.004G086300	AT5G16600	MYB43	**3.456**	<0.001
	Potri.015G033600	AT1G17950	MYB52	**-2.376**	<0.001
	Potri.005G186400	AT1G17950	MYB52	**-2.340**	<0.001
	Potri.012G039400	AT1G17950	MYB52	**-1.495**	<0.001
	Potri.007G134500	AT1G17950	MYB52	**-1.371**	<0.001
	Potri.002G073500	AT1G17950	MYB52	**-1.107**	<0.001
	Potri.005G112000	AT4G38620	MYB4	**-3.359**	<0.001
	Potri.009G134000	AT4G38620	MYB4	**-2.726**	<0.001
	Potri.004G138000	AT4G38620	MYB4	**-2.538**	<0.001

	Potri.004G174400	AT4G38620	MYB4	**-1.762**	<0.001
	Potri.006G221800	AT4G38620	MYB4	**3.180**	<0.001
	Potri.001G112200	AT1G62990	KNAT7	**-2.216**	<0.001
	Potri.013G001000	AT1G09540	MYB61	**-4.555**	<0.001
	Potri.014G111200	AT4G01680	MYB61	**-2.753**	<0.001
	Potri.005G001600	AT1G09540	MYB61	**-2.651**	<0.001
	Potri.007G106100	AT4G33450	MYB69	**-2.327**	<0.001
	Potri.005G063200	AT4G33450	MYB69	**-0.892**	0.001
	Potri.015G129100	AT4G22680	MYB85	**-3.833**	<0.001

Supplement Table S6 DEGs involved in lignin biosynthesis in poplar wood (hybrid T89) in response to severe drought stress

Log_2-fold changes (FC) of up-regulated genes are shown in dark red and log_2-FC of down-regulated genes in dark blue. DEGs are genes with p_{adj} (Bonferroni correction) < 0.05.

Function	Potri.ID	AGI	At.name	log_2-FC	p_{adj}
Phenylpropanoid pathway	Potri.001G036900	AT3G21240	4CL1	-1.992	<0.001
	Potri.003G188500	AT3G21240	4CL3	-2.226	<0.001
	Potri.019G049500	AT1G65060	4CL3	2.383	0.004
	Potri.006G033300	AT2G40890	C3H	-2.213	<0.001
	Potri.016G031100	AT2G40890	C3H	1.924	<0.001
	Potri.016G031000	AT2G40890	C3H	1.998	<0.001
	Potri.013G157900	AT2G30490	C4H	-3.246	<0.001
	Potri.019G130700	AT2G30490	C4H	-1.489	<0.001
	Potri.014G195800	AT5G44070	CAD	-0.559	<0.001
	Potri.006G107900	AT1G29690	CAD1	-0.994	<0.001
	Potri.009G099800	AT4G34050	CCOAMT1	-2.979	<0.001
	Potri.001G304800	AT4G34050	CCOAMT1	-2.115	<0.001
	Potri.001G045500	AT1G15950	CCR2	0.852	0.002
	Potri.001G045400	AT1G80820	CCR2	1.893	0.001
	Potri.012G006400	AT5G54160	COMT	-2.970	<0.001
	Potri.015G003100	AT5G54160	COMT	-2.877	<0.001
	Potri.002G180700	AT5G54160	COMT	-1.971	<0.001
	Potri.002G180600	AT5G54160	COMT	-1.821	<0.001
	Potri.002G180500	AT5G54160	COMT	-1.755	0.002
	Potri.014G106500	AT5G54160	COMT	-1.219	0.032
	Potri.003G059200	AT1G52760	CSE	-2.672	<0.001
	Potri.001G175000	AT1G52760	CSE	-1.559	<0.001
	Potri.005G117500	AT4G36220	F5H	-2.802	<0.001
	Potri.007G016400	AT4G36220	F5H	-1.990	<0.001
	Potri.001G042900	AT5G48930	HCT	-1.802	<0.001
	Potri.003G183900	AT5G48930	HCT	-1.193	0.022
	Potri.018G104800	AT5G48930	HCT	1.132	<0.001
	Potri.018G104700	AT5G48930	HCT	1.340	<0.001
	Potri.010G224200	AT2G37040	PAL	-4.169	<0.001
	Potri.010G224100	AT2G37040	PAL	-4.140	<0.001
	Potri.008G038200	AT2G37040	PAL	-1.754	<0.001
	Potri.006G126800	AT2G37040	PAL	-1.240	<0.001
	Potri.016G091100	AT2G37040	PAL	-1.054	<0.001
Lignin polymerization	Potri.019G088500	AT5G09360	LAC14	2.691	0.005
	Potri.019G088800	AT5G09360	LAC14	2.745	0.003
	Potri.019G088900	AT5G09360	LAC14	2.831	0.005
	Potri.019G088700	AT5G09360	LAC14	3.443	<0.001
	Potri.019G088600	AT5G09360	LAC14	3.872	<0.001

	Potri.006G087100	AT5G60020	LAC17	**-6.238**	<0.001
	Potri.006G087500	AT5G60020	LAC17	**-6.179**	<0.001
	Potri.001G401300	AT5G60020	LAC17	**-5.304**	<0.001
	Potri.001G401100	AT5G60020	LAC17	**-4.860**	<0.001
	Potri.001G184300	AT5G60020	LAC17	**-4.430**	<0.001
	Potri.011G120300	AT5G60020	LAC17	**-4.157**	<0.001
	Potri.001G054600	AT5G60020	LAC17	**-3.988**	<0.001
	Potri.011G120200	AT5G60020	LAC17	**-3.885**	<0.001
	Potri.006G096900	AT2G38080	LAC4/IRX12	**-4.674**	<0.001
	Potri.010G193100	AT2G38080	LAC4/IRX12	**-4.379**	<0.001
	Potri.006G097000	AT2G38080	LAC4/IRX12	**-4.345**	<0.001
	Potri.009G042500	AT2G38080	LAC4/IRX12	**-4.267**	<0.001
	Potri.006G097100	AT2G38080	LAC4/IRX12	**-3.952**	<0.001
	Potri.008G064000	AT2G38080	LAC4/IRX12	**-3.831**	<0.001
	Potri.016G112100	AT2G38080	LAC4/IRX12	**-3.437**	<0.001
	Potri.016G112000	AT2G38080	LAC4/IRX12	**-3.335**	<0.001
	Potri.001G248700	AT2G38080	LAC4/IRX12	**-2.211**	<0.001

Supplement Table S7 DEGs that were classified by MapMan in the category "cell wall" in poplar wood (hybrid T89).

Log_2-fold changes (FC) of up-regulated genes are shown in dark red and log_2-FC of down-regulated genes in dark blue. DEGs are genes with p_{adj} (Bonferroni correction) < 0.05.

MapMan. cell wall category	Potri.ID	AGI	log_2-FC	p_{adj}	At.name
[10.1] cell wall.precursor synthesis	Potri.006G090300	At2g39770	-0.496	0.012	CYT1
[10.1] cell wall.precursor synthesis	Potri.008G006700	At2g39770	-3.338	<0.001	CYT1
[10.1] cell wall.precursor synthesis	Potri.010G198800	At2g39770	-2.947	<0.001	CYT1
[10.1] cell wall.precursor synthesis	Potri.008G060100	At2g39770	-2.196	<0.001	CYT1
[10.1] cell wall.precursor synthesis	Potri.002G077400	At5g52560	-1.482	<0.001	USP
[10.1] cell wall.precursor synthesis	Potri.006G272700	At1g78570	-1.531	0.013	RHM1
[10.1] cell wall.precursor synthesis	Potri.001G112000	At1g63000	-1.272	0.001	UER1
[10.1] cell wall.precursor synthesis	Potri.003G120000	At1g63000	-2.926	<0.001	UER2
[10.1] cell wall.precursor synthesis	Potri.002G037400	At3g54690	1.091	0.003	
[10.1] cell wall.precursor synthesis	Potri.002G061900	At1g79500	-0.907	<0.001	kdsA1
[10.1] cell wall.precursor synthesis	Potri.005G199100	At1g79500	-1.468	<0.001	kdsA1
[10.1] cell wall.precursor synthesis	Potri.004G099100	At1g67070	-2.794	<0.001	DIN9
[10.1] cell wall.precursor synthesis	Potri.014G080300	At2g45790	-2.931	<0.001	PMM
[10.1] cell wall.precursor synthesis	Potri.014G080500	At2g45790	-2.896	<0.001	PMM
[10.1] cell wall.precursor synthesis	Potri.006G070500	At3g10700	-2.984	<0.001	GalAK
[10.1] cell wall.precursor synthesis	Potri.001G343400	At3g01640	-1.383	<0.001	GLCAK
[10.1] cell wall.precursor synthesis	Potri.008G094300	At3g29360	-3.651	<0.001	
[10.1] cell wall.precursor synthesis	Potri.017G092000	At3g29360	-1.374	0.032	
[10.1] cell wall.precursor synthesis	Potri.004G118600	At5g15490	-3.975	<0.001	
[10.1] cell wall.precursor synthesis	Potri.010G159800	At5g15490	-0.980	0.013	
[10.1] cell wall.precursor synthesis	Potri.006G214000	At3g53520	-0.609	<0.001	UXS1
[10.1] cell wall.precursor synthesis	Potri.016G080500	At3g53520	-1.122	<0.001	UXS2
[10.1] cell wall.precursor synthesis	Potri.001G237200	At3g46440	-2.451	<0.001	UXS5
[10.1] cell wall.precursor synthesis	Potri.010G207200	At2g28760	-3.090	<0.001	UXS6
[10.1] cell wall.precursor synthesis	Potri.008G053100	At2g28760	-1.526	<0.001	UXS6
[10.1] cell wall.precursor synthesis	Potri.018G100400	At4g30440	1.142	0.022	GAE1
[10.1] cell wall.precursor synthesis	Potri.002G116800	At1g02000	-1.531	<0.001	GAE2
[10.1] cell wall.precursor synthesis	Potri.012G128200	At1g02000	-2.441	<0.001	GAE2
[10.1] cell wall.precursor synthesis	Potri.014G068400	At1g02000	-2.838	<0.001	GAE2
[10.1] cell wall.precursor synthesis	Potri.002G146500	At4g00110	-2.607	<0.001	GAE3
[10.1] cell wall.precursor synthesis	Potri.003G114600	At4g00110	-0.737	0.02	GAE3
[10.1] cell wall.precursor synthesis	Potri.017G059100	At3g23820	-3.834	<0.001	GAE6
[10.1] cell wall.precursor synthesis	Potri.006G022000	At1g30620	1.744	0.002	MUR4
[10.1] cell wall.precursor synthesis	Potri.003G073500	At1g17270	-2.750	<0.001	
[10.2] cell wall.cellulose synthesis	Potri.005G087500	At5g64740	-0.850	0.045	CESA6
[10.2] cell wall.cellulose synthesis	Potri.002G257900	At5g44030	-3.059	<0.001	CESA4
[10.2] cell wall.cellulose synthesis	Potri.002G066600	At2g21770	-0.951	0.003	CESA9

[10.2] cell wall.cellulose synthesis	Potri.013G019800	At2g21770	**-1.391**	<0.001	CESA10
[10.2] cell wall.cellulose synthesis	Potri.004G059600	At4g18780	**-3.724**	<0.001	CESA8
[10.2] cell wall.cellulose synthesis	Potri.011G069600	At4g18780	**-3.925**	<0.001	CESA8
[10.2] cell wall.cellulose synthesis	Potri.006G052600	At5g05170	**-2.601**	<0.001	CESA3
[10.2] cell wall.cellulose synthesis	Potri.006G181900	At5g17420	**-3.338**	<0.001	CESA7
[10.2] cell wall.cellulose synthesis	Potri.018G103900	At5g17420	**-4.022**	<0.001	CESA8
[10.2] cell wall.cellulose synthesis	Potri.004G208800	At4g38190	**1.639**	0.011	CSLD4
[10.2] cell wall.cellulose synthesis	Potri.006G004232	At1g55850	**0.741**	0.044	CSLE1
[10.2] cell wall.cellulose synthesis	Potri.006G004300	At1g55850	**-1.454**	0.014	CSLE1
[10.2] cell wall.cellulose synthesis	Potri.003G142300	At4g23990	**0.832**	0.018	CSLG3
[10.2] cell wall.cellulose synthesis	Potri.003G142400	At4g23990	**0.992**	0.022	CSLG3
[10.2] cell wall.cellulose synthesis	Potri.001G136200	At3g03050	**-0.999**	0.015	CSLD3
[10.2] cell wall.cellulose synthesis	Potri.003G097100	At3g03050	**-1.540**	<0.001	CSLD
[10.2] cell wall.cellulose synthesis	Potri.002G200300	At1g02730	**-2.239**	<0.001	CSLD
[10.2] cell wall.cellulose synthesis	Potri.014G125100	At1g02730	**-2.707**	<0.001	CSLD5
[10.2] cell wall.cellulose synthesis	Potri.004G117200	At5g15630	**-4.954**	<0.001	COBL4
[10.2] cell wall.cellulose synthesis	Potri.015G060100	At5g15630	**-3.959**	<0.001	COBL4
[10.2] cell wall.cellulose synthesis	Potri.011G135200	At3g20580	**1.627**	0.008	COBL10
[10.2] cell wall.cellulose synthesis	Potri.014G125300	At4g16120	**-1.644**	<0.001	COBL7
[10.2] cell wall.cellulose synthesis	Potri.009G149700	At5g22740	**0.921**	0.032	CSLA2
[10.2] cell wall.cellulose synthesis	Potri.018G009300	At4g31590	**-1.407**	<0.001	CSLC5
[10.2] cell wall.cellulose synthesis	Potri.001G078900	At5g49720	**-2.119**	<0.001	KOR1
[10.2] cell wall.cellulose synthesis	Potri.003G151700	At5g49720	**-2.530**	<0.001	KOR1
[10.2] cell wall.cellulose synthesis	Potri.001G031500	At5g22130	**-0.758**	0.001	PNT1
[10.2] cell wall.cellulose synthesis	Potri.008G026400	At5g03760	**-3.954**	<0.001	CSLA9
[10.2] cell wall.cellulose synthesis	Potri.010G234100	At5g03760	**-3.843**	<0.001	CSLA9
[10.3] cell wall.hemicellulose synthesis	Potri.002G256200	At2g20370	**1.316**	0.003	MUR3
[10.3] cell wall.hemicellulose synthesis	Potri.009G006500	At2g28110	**-2.849**	<0.001	FRA8
[10.3] cell wall.hemicellulose synthesis	Potri.001G416800	At5g54690	**-3.383**	<0.001	GAUT12
[10.3] cell wall.hemicellulose synthesis	Potri.011G132600	At5g54690	**-3.424**	<0.001	GAUT12
[10.3] cell wall.hemicellulose synthesis	Potri.002G132900	At1g19300	**-4.012**	<0.001	GATL1
[10.3] cell wall.hemicellulose synthesis	Potri.014G040300	At1g19300	**-4.116**	<0.001	GATL1
[10.3] cell wall.hemicellulose synthesis	Potri.006G131000	At2g37090	**-4.822**	<0.001	IRX9
[10.3] cell wall.hemicellulose synthesis	Potri.016G086400	At2g37090	**-3.048**	<0.001	IRX9
[10.3] cell wall.hemicellulose synthesis	Potri.005G061600	At3g18660	**-3.773**	<0.001	GUX1
[10.3] cell wall.hemicellulose synthesis	Potri.007G107200	At3g18660	**-3.617**	<0.001	GUX
[10.3] cell wall.hemicellulose synthesis	Potri.014G029900	At4g33330	**-2.550**	<0.001	GUX2
[10.3] cell wall.hemicellulose synthesis	Potri.013G067400	At3g45400	**-3.048**	<0.001	
[10.3] cell wall.hemicellulose synthesis	Potri.019G044600	At3g45400	**-0.942**	<0.001	
[10.3] cell wall.hemicellulose synthesis	Potri.019G049800	At5g16890	**-0.425**	<0.001	
[10.3] cell wall.hemicellulose synthesis	Potri.018G125000	At5g20260	**1.103**	0.003	
[10.3] cell wall.hemicellulose synthesis	Potri.006G064800	At5g20260	**-1.578**	<0.001	
[10.3] cell wall.hemicellulose synthesis	Potri.018G124900	At5g20260	**-2.708**	<0.001	
[10.3] cell wall.hemicellulose synthesis	Potri.003G191200	At2g03220	**-1.263**	0.001	MUR2
[10.3] cell wall.hemicellulose synthesis	Potri.003G191301	At2g03220	**-0.542**	<0.001	MUR2
[10.3] cell wall.hemicellulose synthesis	Potri.003G191401	At2g03220	**-1.294**	<0.001	MUR2

[10.3] cell wall.hemicellulose synthesis	Potri.010G040400	At5g62620	-1.576	<0.001	
[10.3] cell wall.hemicellulose synthesis	Potri.012G072400	At5g62620	-0.527	0.005	
[10.4] cell wall.pectin synthesis	Potri.002G151400	At3g61130	-0.931	<0.001	GAUT1
[10.4] cell wall.pectin synthesis	Potri.014G073800	At3g61130	-0.554	0.032	GAUT1
[10.4] cell wall.pectin synthesis	Potri.002G166000	At4g01220	-0.758	0.003	
[10.4] cell wall.pectin synthesis	Potri.002G246500	At3g25140	-1.126	0.001	GAUT8
[10.5] cell wall.cell wall proteins	Potri.005G144900	At4g37450	-1.208	0.045	AGP18
[10.5] cell wall.cell wall proteins	Potri.009G092300	At4g37450	-2.444	<0.001	AGP18
[10.5] cell wall.cell wall proteins	Potri.017G050200	At5g64310	5.224	<0.001	AGP1
[10.5] cell wall.cell wall proteins	Potri.017G050300	At5g64310	2.220	0.001	AGP1
[10.5] cell wall.cell wall proteins	Potri.001G004100	At5g56540	-0.782	0.002	AGP14
[10.5] cell wall.cell wall proteins	Potri.003G220901	At5g56540	-1.196	0.001	AGP14
[10.5] cell wall.cell wall proteins	Potri.013G057500	At5g56540	-4.494	<0.001	AGP14
[10.5] cell wall.cell wall proteins	Potri.019G035500	At5g56540	-0.869	0.033	AGP14
[10.5] cell wall.cell wall proteins	Potri.010G132500	At1g68725	-4.216	<0.001	AGP19
[10.5] cell wall.cell wall proteins	Potri.003G136600	At3g61640	1.396	0.01	AGP20
[10.5] cell wall.cell wall proteins	Potri.014G094800	At3g61640	-3.604	<0.001	AGP20
[10.5] cell wall.cell wall proteins	Potri.018G078801	At5g53250	3.404	0.001	AGP22
[10.5] cell wall.cell wall proteins	Potri.002G207500	At2g47930	-3.523	<0.001	AGP26
[10.5] cell wall.cell wall proteins	Potri.002G101101	At4g40090	1.865	0.015	AGP3
[10.5] cell wall.cell wall proteins	Potri.013G120600	At2g20520	-2.553	0.001	FLA6
[10.5] cell wall.cell wall proteins	Potri.006G174900	At3g46550	-0.749	<0.001	SOS5
[10.5] cell wall.cell wall proteins	Potri.018G097000	At3g46550	-1.567	<0.001	SOS5
[10.5] cell wall.cell wall proteins	Potri.006G200300	At5g06390	-2.441	<0.001	FLA17
[10.5] cell wall.cell wall proteins	Potri.008G012400	At5g06390	-3.793	<0.001	FLA17
[10.5] cell wall.cell wall proteins	Potri.010G244900	At5g06390	-3.236	<0.001	FLA17
[10.5] cell wall.cell wall proteins	Potri.012G006200	At5g06390	-3.564	<0.001	FLA17
[10.5] cell wall.cell wall proteins	Potri.016G066500	At5g06390	-1.332	<0.001	FLA17
[10.5] cell wall.cell wall proteins	Potri.019G008400	At5g06390	-3.485	<0.001	FLA17
[10.5] cell wall.cell wall proteins	Potri.014G071700	At3g60900	-0.956	0.007	FLA10
[10.5] cell wall.cell wall proteins	Potri.001G320800	At5g03170	-6.057	<0.001	FLA11
[10.5] cell wall.cell wall proteins	Potri.006G129200	At5g03170	-4.151	<0.001	FLA11
[10.5] cell wall.cell wall proteins	Potri.009G012100	At5g03170	-6.302	<0.001	FLA11
[10.5] cell wall.cell wall proteins	Potri.012G015000	At5g03170	-7.048	<0.001	FLA11
[10.5] cell wall.cell wall proteins	Potri.012G127900	At5g03170	-5.660	<0.001	FLA11
[10.5] cell wall.cell wall proteins	Potri.013G151366	At5g03170	-7.815	<0.001	FLA11
[10.5] cell wall.cell wall proteins	Potri.015G013300	At5g03170	-5.951	<0.001	FLA11
[10.5] cell wall.cell wall proteins	Potri.015G129400	At5g03170	-5.255	<0.001	FLA11
[10.5] cell wall.cell wall proteins	Potri.016G088700	At5g03170	-4.077	<0.001	FLA11
[10.5] cell wall.cell wall proteins	Potri.019G120900	At5g03170	-5.406	<0.001	FLA11
[10.5] cell wall.cell wall proteins	Potri.019G121100	At5g03170	-6.677	<0.001	FLA11
[10.5] cell wall.cell wall proteins	Potri.019G121200	At5g03170	-7.012	<0.001	FLA11
[10.5] cell wall.cell wall proteins	Potri.019G121300	At5g03170	-7.676	<0.001	FLA11
[10.5] cell wall.cell wall proteins	Potri.019G122800	At5g03170	-6.652	<0.001	FLA11
[10.5] cell wall.cell wall proteins	Potri.019G123100	At5g03170	-6.474	<0.001	FLA11
[10.5] cell wall.cell wall proteins	Potri.019G123200	At5g03170	-7.417	<0.001	FLA11

[10.5] cell wall.cell wall proteins	Potri.004G210600	At5g60490	-7.680	<0.001	FLA12
[10.5] cell wall.cell wall proteins	Potri.009G012200	At5g60490	-7.517	<0.001	FLA12
[10.5] cell wall.cell wall proteins	Potri.013G014200	At5g60490	-7.994	<0.001	FLA12
[10.5] cell wall.cell wall proteins	Potri.013G151300	At5g60490	-7.012	<0.001	FLA12
[10.5] cell wall.cell wall proteins	Potri.013G151432	At5g60490	-7.662	<0.001	FLA12
[10.5] cell wall.cell wall proteins	Potri.013G151500	At5g60490	-7.300	<0.001	FLA12
[10.5] cell wall.cell wall proteins	Potri.019G120800	At5g60490	-5.415	<0.001	FLA12
[10.5] cell wall.cell wall proteins	Potri.019G122600	At5g60490	-6.505	<0.001	FLA12
[10.5] cell wall.cell wall proteins	Potri.019G123000	At5g60490	-5.380	<0.001	FLA12
[10.5] cell wall.cell wall proteins	Potri.002G223300	At2g04780	-2.779	<0.001	FLA7
[10.5] cell wall.cell wall proteins	Potri.014G162900	At2g04780	-3.385	<0.001	FLA7
[10.5] cell wall.cell wall proteins	Potri.001G302500	At4g06744	1.563	0.001	
[10.5] cell wall.cell wall proteins	Potri.014G024300	At4g06744	2.444	<0.001	
[10.5] cell wall.cell wall proteins	Potri.009G098700	At4g06744	-1.269	0.031	
[10.5] cell wall.cell wall proteins	Potri.018G035100	At4g18670	-1.582	0.002	
[10.5] cell wall.cell wall proteins	Potri.007G139200	At4g28380	-2.674	<0.001	
[10.5] cell wall.cell wall proteins	Potri.006G152800	At4g29240	0.818	0.049	
[10.5] cell wall.cell wall proteins	Potri.006G081200	At1g62440	-1.132	0.009	LRX2
[10.5] cell wall.cell wall proteins	Potri.008G211800	At2g42800	-1.262	0.025	RLP29
[10.5] cell wall.cell wall proteins	Potri.014G102501	At2g46630	2.757	0.001	
[10.5] cell wall.cell wall proteins	Potri.001G155000	At4g16790	-1.555	0.005	
[10.5] cell wall.cell wall proteins	Potri.013G131600	At4g28300	1.091	0.01	
[10.5] cell wall.cell wall proteins	Potri.019G051700	At5g16510	0.451	0.001	
[10.5] cell wall.cell wall proteins	Potri.004G117800	At5g15650	1.857	0.017	RGP2
[10.5] cell wall.cell wall proteins	Potri.017G099100	At5g15650	3.849	<0.001	RGP2
[10.5] cell wall.cell wall proteins	Potri.010G156700	At3g08900	0.431	0.045	RGP3
[10.5] cell wall.cell wall proteins	Potri.015G060300	At3g08900	-1.083	0.044	RGP3
[10.5] cell wall.cell wall proteins	Potri.013G144200	At2g20870	-2.027	<0.001	
[10.5] cell wall.cell wall proteins	Potri.012G097000	At3g48530	1.732	<0.001	KING1
[10.5] cell wall.cell wall proteins	Potri.015G094700	At3g48530	1.893	<0.001	KING1
[10.5] cell wall.cell wall proteins	Potri.002G168500	At3g58800	-0.850	0.001	
[10.6] cell wall.degradation	Potri.002G023900	At1g19940	-3.286	<0.001	GH9B5
[10.6] cell wall.degradation	Potri.005G237700	At1g19940	-3.065	<0.001	GH9B5
[10.6] cell wall.degradation	Potri.019G037400	At5g20950	-1.197	0.038	
[10.6] cell wall.degradation	Potri.006G062200	At5g34940	-3.476	<0.001	GUS3
[10.6] cell wall.degradation	Potri.018G024200	At5g34940	-1.308	<0.001	GUS3
[10.6] cell wall.degradation	Potri.018G121500	At5g34940	-1.697	<0.001	GUS3
[10.6] cell wall.degradation	Potri.006G027700	At3g10740	1.146	<0.001	ASD1
[10.6] cell wall.degradation	Potri.006G029900	At3g10740	1.099	<0.001	ASD1
[10.6] cell wall.degradation	Potri.016G025500	At3g10740	-2.202	<0.001	ASD1
[10.6] cell wall.degradation	Potri.009G006900	At2g28100	-1.789	<0.001	FUC1
[10.6] cell wall.degradation	Potri.001G206800	At5g64570	-5.090	<0.001	XYL4
[10.6] cell wall.degradation	Potri.003G022900	At5g64570	-4.938	<0.001	XYL4
[10.6] cell wall.degradation	Potri.008G108100	At5g49360	-2.034	0.007	BXL1
[10.6] cell wall.degradation	Potri.002G197200	At1g02640	-4.316	<0.001	BXL2
[10.6] cell wall.degradation	Potri.014G122200	At1g02640	-4.545	<0.001	BXL2

[10.6] cell wall.degradation	Potri.002G113132	At1g58370	-3.777	<0.001	RXF12
[10.6] cell wall.degradation	Potri.002G094000	At1g78060	-1.301	0.009	
[10.6] cell wall.degradation	Potri.001G089100	At5g10560	-1.003	0.001	
[10.6] cell wall.degradation	Potri.006G109900	At5g01930	-3.981	<0.001	
[10.6] cell wall.degradation	Potri.016G138600	At5g01930	-4.043	<0.001	
[10.6] cell wall.degradation	Potri.002G033501	At5g66460	1.331	0.013	
[10.6] cell wall.degradation	Potri.008G182200	At1g67750	-3.264	<0.001	
[10.6] cell wall.degradation	Potri.010G051800	At1g67750	-3.802	<0.001	
[10.6] cell wall.degradation	Potri.002G190600	At1g02460	-3.578	<0.001	
[10.6] cell wall.degradation	Potri.014G115700	At1g02460	-1.603	0.014	
[10.6] cell wall.degradation	Potri.018G062700	At1g19170	-1.876	<0.001	
[10.6] cell wall.degradation	Potri.011G159000	At1g23460	-3.620	<0.001	
[10.6] cell wall.degradation	Potri.008G100500	At1g48100	-3.675	0.002	
[10.6] cell wall.degradation	Potri.010G152000	At1g48100	-2.545	0.003	
[10.6] cell wall.degradation	Potri.008G189200	At1g60590	-1.674	0.002	
[10.6] cell wall.degradation	Potri.010G042100	At1g60590	-1.380	0.023	
[10.6] cell wall.degradation	Potri.001G463000	At1g70500	-3.930	<0.001	
[10.6] cell wall.degradation	Potri.007G145300	At2g43890	1.171	0.038	
[10.6] cell wall.degradation	Potri.006G052700	At3g07970	-1.450	0.007	QRT2
[10.6] cell wall.degradation	Potri.010G005500	At3g16850	-1.417	<0.001	
[10.6] cell wall.degradation	Potri.006G139500	At3g42950	-2.190	<0.001	
[10.6] cell wall.degradation	Potri.006G122000	At3g53190	-2.566	<0.001	
[10.6] cell wall.degradation	Potri.006G214400	At3g55140	2.077	<0.001	
[10.6] cell wall.degradation	Potri.008G048600	At3g55140	0.837	0.009	
[10.6] cell wall.degradation	Potri.016G080600	At3g55140	0.883	<0.001	
[10.6] cell wall.degradation	Potri.016G051200	At3g57790	-1.014	<0.001	
[10.6] cell wall.degradation	Potri.003G131700	At3g61490	-2.682	<0.001	
[10.6] cell wall.degradation	Potri.001G052300	At4g13710	-3.637	<0.001	
[10.6] cell wall.degradation	Potri.002G238800	At4g13710	-2.939	<0.001	
[10.6] cell wall.degradation	Potri.003G175900	At4g13710	-5.652	<0.001	
[10.6] cell wall.degradation	Potri.014G178100	At4g13710	-4.217	<0.001	
[10.6] cell wall.degradation	Potri.009G169100	At4g18180	1.408	<0.001	
[10.6] cell wall.degradation	Potri.001G100000	At4g23500	-1.725	<0.001	
[10.6] cell wall.degradation	Potri.006G196400	At4g24780	-2.995	<0.001	
[10.6] cell wall.degradation	Potri.012G091300	At4g24780	-3.588	<0.001	
[10.6] cell wall.degradation	Potri.012G091500	At4g24780	-3.804	<0.001	
[10.6] cell wall.degradation	Potri.007G105800	At4g33440	1.596	<0.001	
[10.6] cell wall.degradation	Potri.008G032700	At5g04310	-5.064	<0.001	
[10.6] cell wall.degradation	Potri.010G229000	At5g04310	-1.247	0.049	
[10.6] cell wall.degradation	Potri.010G008600	At5g49215	-1.021	0.003	
[10.6] cell wall.degradation	Potri.015G087800	At5g63180	-3.509	<0.001	
[10.6] cell wall.degradation	Potri.010G040800	At1g70370	-2.118	<0.001	PG2
[10.6] cell wall.degradation	Potri.010G040900	At1g70370	-2.236	<0.001	PG2
[10.6] cell wall.degradation	Potri.008G190000	At1g70370	-1.677	<0.001	PG2
[10.6] cell wall.degradation	Potri.012G079200	At1g70370	-2.582	<0.001	PG2
[10.6] cell wall.degradation	Potri.002G110000	At1g09890	-1.585	0.003	

[10.6] cell wall.degradation	Potri.002G110200	At1g09890	**-3.262**	<0.001	
[10.6] cell wall.degradation	Potri.006G218500	At2g22620	**-3.438**	<0.001	
[10.6] cell wall.degradation	Potri.006G253601	At2g22620	**-4.184**	<0.001	
[10.6] cell wall.degradation	Potri.014G004500	At2g22620	**-6.432**	<0.001	
[10.7] cell wall.modification	Potri.006G086100	At2g37640	**1.188**	0.031	EXPA3
[10.7] cell wall.modification	Potri.018G031901	At4g30380	**-1.174**	<0.001	
[10.7] cell wall.modification	Potri.018G041300	At4g30380	**-0.827**	0.042	
[10.7] cell wall.modification	Potri.010G167200	At1g69530	**-2.522**	0.001	EXPA1
[10.7] cell wall.modification	Potri.017G140000	At3g03220	**-1.459**	<0.001	EXPA13
[10.7] cell wall.modification	Potri.001G240900	At2g39700	**-4.951**	<0.001	EXPA4
[10.7] cell wall.modification	Potri.009G031800	At2g39700	**-2.840**	<0.001	EXPA4
[10.7] cell wall.modification	Potri.010G202500	At2g39700	**-1.606**	0.009	EXPA4
[10.7] cell wall.modification	Potri.019G057500	At2g40610	**-1.623**	0.036	EXPA8
[10.7] cell wall.modification	Potri.013G134300	At4g28250	**-2.890**	<0.001	EXPB3
[10.7] cell wall.modification	Potri.019G101900	At4g28250	**-2.976**	<0.001	EXPB3
[10.7] cell wall.modification	Potri.007G083400	At4g38400	**3.738**	<0.001	EXLA2
[10.7] cell wall.modification	Potri.009G141400	At4g38400	**3.720**	<0.001	EXLA2
[10.7] cell wall.modification	Potri.001G147200	At4g17030	**3.365**	0.001	EXLB1
[10.7] cell wall.modification	Potri.001G151500	At4g17030	**10.056**	<0.001	EXLB1
[10.7] cell wall.modification	Potri.003G083200	At4g17030	**9.114**	<0.001	EXLB1
[10.7] cell wall.modification	Potri.003G087000	At4g17030	**3.779**	<0.001	EXLB1
[10.7] cell wall.modification	Potri.006G179300	At2g18660	**3.218**	<0.001	PNP-A
[10.7] cell wall.modification	Potri.018G029100	At2g18660	**1.609**	0.017	PNP-A
[10.7] cell wall.modification	Potri.006G155000	At2g18660	**-2.065**	<0.001	PNP-A
[10.7] cell wall.modification	Potri.009G083800	At2g14620	**-2.487**	<0.001	XTH10
[10.7] cell wall.modification	Potri.002G236200	At3g23730	**1.492**	0.004	XTH16
[10.7] cell wall.modification	Potri.010G102300	At1g14720	**0.913**	0.007	XTH28
[10.7] cell wall.modification	Potri.003G097300	At1g32170	**1.582**	<0.001	XTH30
[10.7] cell wall.modification	Potri.014G140300	At5g13870	**-2.210**	<0.001	XTH5
[10.7] cell wall.modification	Potri.019G125000	At4g03210	**-3.969**	<0.001	XTH9
[10.7] cell wall.modification	Potri.018G094800	At5g57560	**1.872**	0.049	XTH22
[10.7] cell wall.modification	Potri.005G007200	At4g25810	**1.817**	0.038	XTH23
[10.7] cell wall.modification	Potri.006G071200	At4g25810	**3.871**	<0.001	XTH23
[10.7] cell wall.modification	Potri.013G005700	At4g25810	**2.124**	0.007	XTH23
[10.7] cell wall.modification	Potri.018G094900	At4g25810	**1.987**	0.003	XTH23
[10.7] cell wall.modification	Potri.014G115000	At1g10550	**-4.102**	<0.001	XTH33
[10.8] cell wall.pectin-esterases	Potri.011G025400	At1g11580	**1.694**	0.004	PMEPCRA
[10.8] cell wall.pectin-esterases	Potri.016G017700	At1g05310	**-4.011**	<0.001	
[10.8] cell wall.pectin-esterases	Potri.007G015700	At5g19730	**1.613**	0.003	
[10.8] cell wall.pectin-esterases	Potri.003G072800	At3g14310	**2.717**	<0.001	PME3
[10.8] cell wall.pectin-esterases	Potri.014G011300	At3g14310	**1.497**	0.019	PME3
[10.8] cell wall.pectin-esterases	Potri.018G051400	At3g14310	**2.482**	<0.001	PME3
[10.8] cell wall.pectin-esterases	Potri.001G162400	At3g14310	**-3.602**	<0.001	PME3
[10.8] cell wall.pectin-esterases	Potri.006G134500	At4g33220	**-2.997**	<0.001	PME44
[10.8] cell wall.pectin-esterases	Potri.014G127000	At1g02810	**-4.289**	<0.001	
[10.8] cell wall.pectin-esterases	Potri.005G022700	At3g05620	**3.062**	<0.001	

[10.8] cell wall.pectin-esterases	Potri.013G013200	At3g05620	**1.462**	0.018	
[10.8] cell wall.pectin-esterases	Potri.010G247700	At3g10720	**2.167**	0.007	
[10.8] cell wall.pectin-esterases	Potri.012G014500	At3g49220	**-1.980**	<0.001	
[10.8] cell wall.pectin-esterases	Potri.005G001500	At3g05910	**0.994**	0.012	
[10.8] cell wall.pectin-esterases	Potri.013G000900	At3g05910	**-3.732**	<0.001	
[10.8] cell wall.pectin-esterases	Potri.006G084900	At3g09410	**-0.685**	0.003	
[10.8] cell wall.pectin-esterases	Potri.014G110900	At3g62060	**0.783**	0.015	
[10.8] cell wall.pectin-esterases	Potri.003G046200	At4g19410	**2.479**	<0.001	
[10.8] cell wall.pectin-esterases	Potri.012G142300	At5g23870	**-3.174**	<0.001	
[10.8] cell wall.pectin-esterases	Potri.010G004400	At5g26670	**-2.433**	<0.001	
[10.8] cell wall.pectin-esterases	Potri.004G233900	At5g45280	**3.124**	<0.001	
[10.8] cell wall.pectin-esterases	Potri.015G127800	At3g47400	**-1.611**	<0.001	
[10.8] cell wall.pectin-esterases	Potri.002G202500	At4g02320	**1.859**	0.002	
[10.8] cell wall.pectin-esterases	Potri.007G068300	At4g00872	**-4.394**	<0.001	
[10.8] cell wall.pectin-esterases	Potri.014G177700	At5g48450	**-0.790**	0.041	SKS3

Supplement Table S8 Results of the principle component analyses (PCA) for ABA core signaling (ABA_CS) and transcription factors regulating the secondary cell wall formation cascade (SCW_TF)

The PCAs for ABA_CS and for SCW_TF were conducted with the transcript abundances of genes involved ABA signaling and the transcription factors of the SCW cascade (Supplements Table S3, S4) and yielded one significant PC, each. Significant PCs were determined by Scree plots and broken stick analyses.

Replicate	Treatment	ABA_CS	SCW_TF
1	control	-5.9465	8.1335
5	control	-5.6194	7.2184
12	control	-7.6682	6.9142
16	control	-5.0012	5.0124
22	control	-5.0261	3.4004
29	control	-3.3553	1.6373
9	severe drought	5.6207	-4.6481
14	severe drought	4.3192	-4.7406
19	severe drought	8.949	-6.2865
21	severe drought	2.4431	-5.2014
24	severe drought	4.0833	-5.0289
27	severe drought	7.2014	-6.4107
Explained Variance		64%	75%

Supplement Table S9 Mass transitions and corresponding conditions for identification of phytohormones shown in Table 2

MRM Transitions		Analyte	DP [declustering potential]	EP [entrance potential]	CE [collision energy]
Q1	Q3				
137	93	SA	-25	-6	-20
141	97	D_4-SA	-25	-6	-22
174	130	IAA	-35	-9	-14
179	135	D_5-IAA	-35	-9	-14
209	59	JA	-30	-4.5	-24
214	62	D_5-JA	-35	-8.5	-24
239	59	12-COOH-JA	-35	-9	-28
263	153	ABA	-35	-4	-14
293	179	D_6-ABA	-80	-10	-42
305	97	12-HSO_4-JA	-30	-4	-32
387	59	12-OH-Glc-JA	-85	-9	-52
425	263	ABA-GE	-30	-10	-16

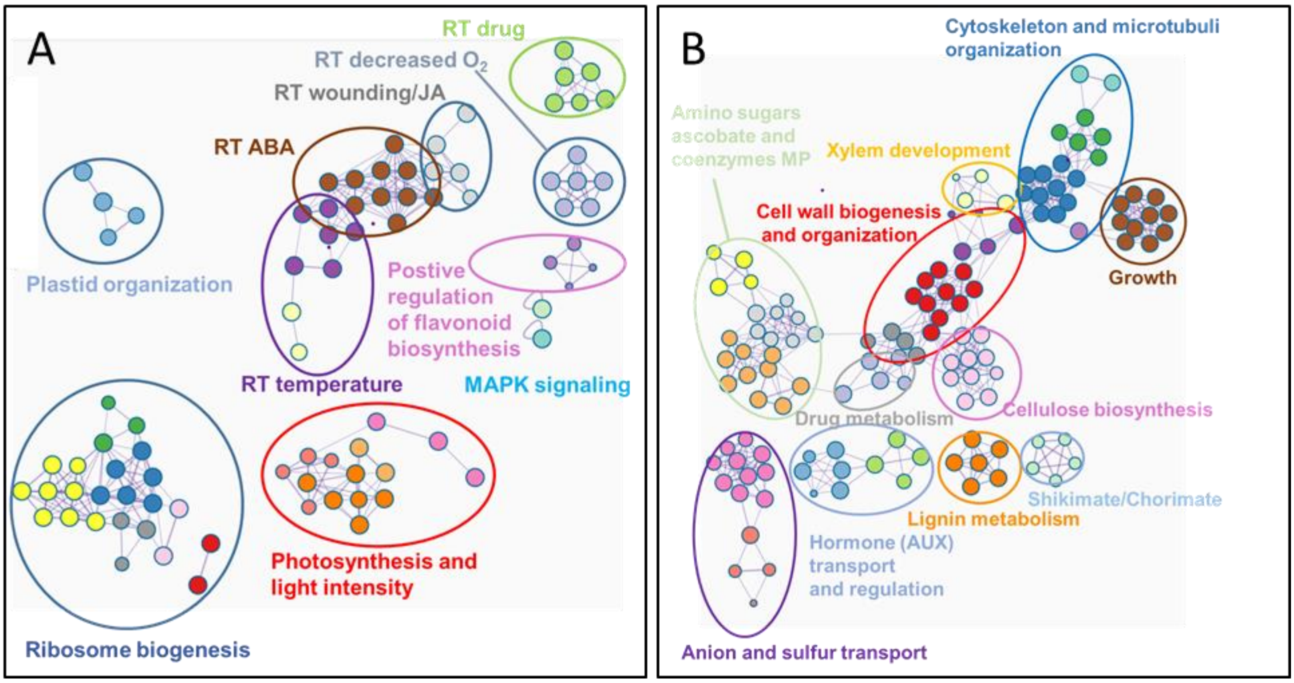

Supplement Figure S1 GO terms enriched with up- (A) and down-(B) regulated genes in poplar wood (hybrid T89) in response to severe drought treatment

A. Clusters of GO terms obtained for 2155 up-regulated genes ($p < 0.05$, $\log_2 > 1$, genes with count mean >50 were included), abbreviation: RT, response to
B. Clusters of GO terms obtained for 2170 down-regulated genes ($p < 0.05$, $\log_2 < -1$, genes with count mean >50 were included)

Figures were produced, uploading the best matches (AGIs) of the poplar DEGs in Metascape [1]. Clusters containing similar GO terms are indicated by the same color.

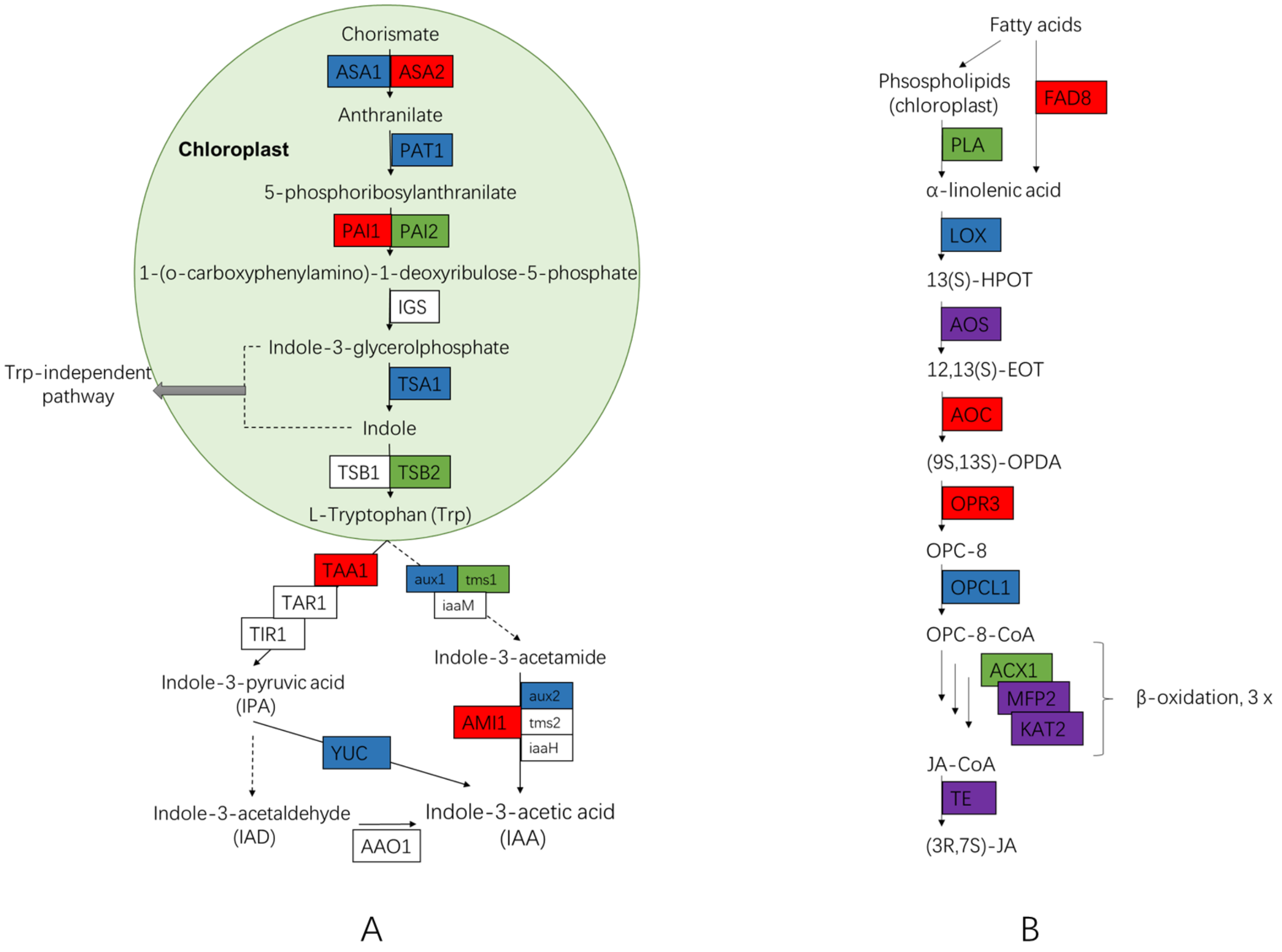

Supplement Figure S2 Transcriptional regulation of genes involved in IAA (A) and JA (B) biosynthesis pathways

A. The pathway of IAA biosynthetic pathway was modified after Mano&Nemoto (2012) [2]. Tryptophan (Trp) synthetic pathway took place in the chloroplast, as shown in the big light green circle. The IAA is synthesized via Trp-independent way (not well understood yet) and Trp-dependent way. Abbreviations: ASA: anthranilate synthase, PAT: phosphoribosylanthranilate transferase, PAI: phosphoribosylanthranilate isomerase, IGS: indole-3-glycerol phosphate synthase, TSA: Trp synthase α, TSB: Trp synthase β, TAA: tryptophan aminotransferase of Arabidopsis, TAR: tryptophan aminotransferase related, TIR: transport inhibitor response, YUC: YUCCA (flavin-containing monooxygenase), AMI: amidase, TMS: tryptophan-2-monooxygenase, AAO: aldehyde oxidase.

B. The figure was modified after Kombrink (2012) [3]. Abbriviations: PLA: phospholipases, LOX: 13-lipoxygenase, AOS: allene oxide synthase, AOC: allene oxide cyclase, OPR3: OPDA reductase 3, OPCL1: OPC-8:CoA ligase, ACX: acyl-CoA oxidase, MFP: multifunctional protein, KAT: L-3-ketoacyl CoA thiolase, TE: thioesterase.

Color code: White: gene not detected. Green: gene not significantly affected. Red: gene significantly up-regulated. Darkblue: gene significantly down-regulated. Please note that poplar often contains several homologs that match one *Arabidopsis thaliana* locus.

Regulation of relevant genes were shown Supplement Table S4.

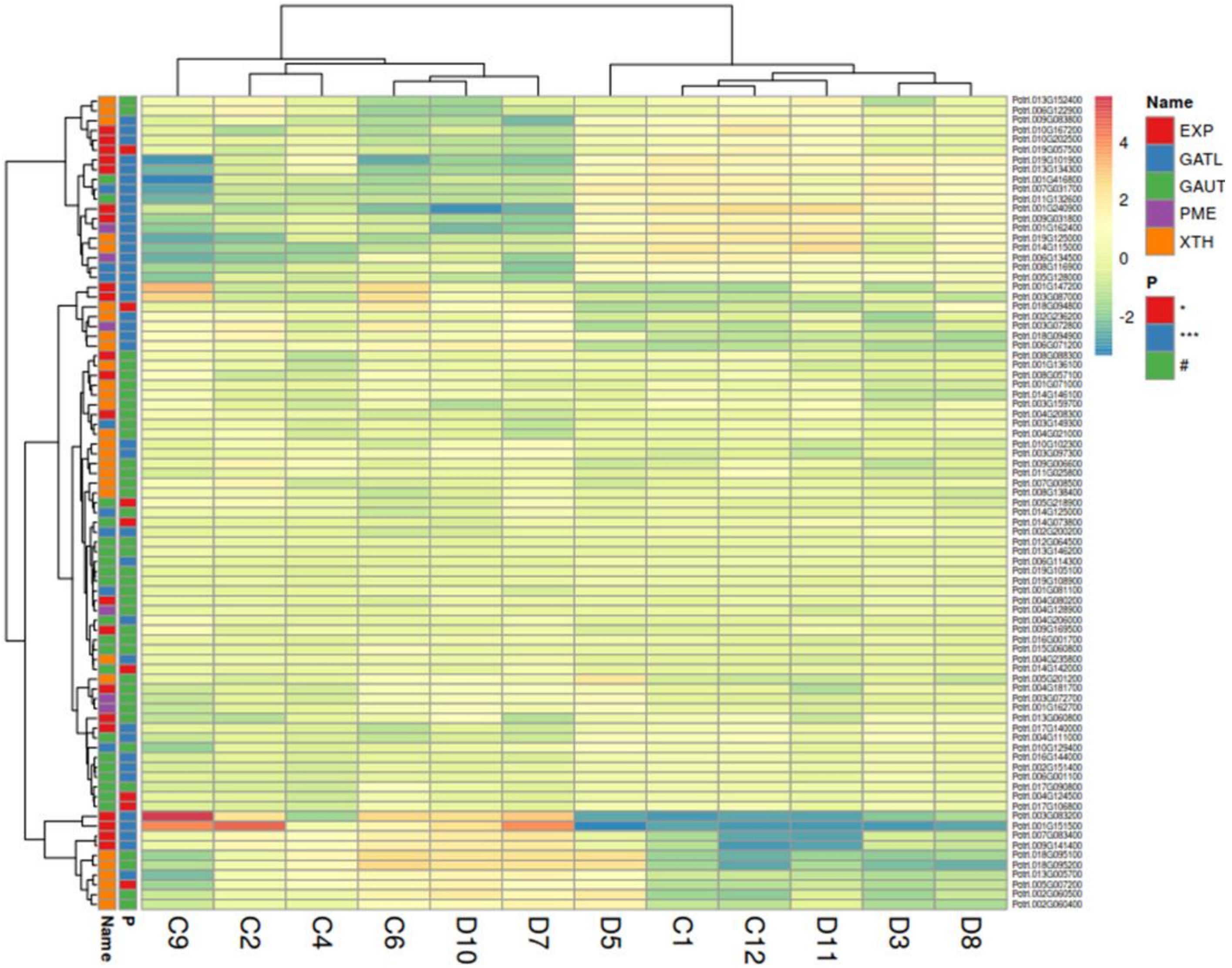

Supplement Figure S3 Hierarchical cluster analysis of genes in poplar wood (hybrid T89) related to hemicellulose formation.

Transcript abundances of genes annotated as EXP, expansin and expansin-like; GATL, galacturonosyltransferase-like; GAUT, galacturonosyltransferase; PME, pectin methyl esterase; XTH, xyloglucan endotransglucosylase/hydrolase were retrieved from Supplemental table S1, and subjected to cluster analysis after transformation [ln(x+1)] using Ward and Euclidian distance. P indicates p-values for the comparison of means of drought-stressed with non-stressed plants: * $p < 0.05$, *** $p < 0.001$, # not significant. C1, C2, C4, C6, C9, and C12 are control samples and D11, D3, D5, D8, D7 and D10 are samples collected from drought-treated plants. Two main clusters were formed. However, control and drought samples were not clearly separated. The heatmap was drawn using ClustVis [4].

Reference

1. Zhou, Y.; Zhou, B.; Pache, L.; Chang, M.; Khodabakhshi, A.H.; Tanaseichuk, O.; Benner, C.; Chanda, S.K. Metascape Provides a Biologist-Oriented Resource for the Analysis of Systems-Level Datasets. *Nature Communications* **2019**, *10*, 1523, doi:10.1038/s41467-019-09234-6.
2. Mano, Y.; Nemoto, K. The Pathway of Auxin Biosynthesis in Plants. *Journal of Experimental Botany* **2012**, *63*, 2853–2872, doi:10.1093/jxb/ers091.
3. Kombrink, E. Chemical and Genetic Exploration of Jasmonate Biosynthesis and Signaling Paths. *Planta* **2012**, *236*, 1351–1366, doi:10.1007/s00425-012-1705-z.
4. Metsalu, T.; Vilo, J. ClustVis: A Web Tool for Visualizing Clustering of Multivariate Data Using Principal Component Analysis and Heatmap. *Nucleic Acids Res* **2015**, *43*, W566-570, doi:10.1093/nar/gkv468.
